# Polysiloxane-Based Side Chain Liquid Crystal Polymers: From Synthesis to Structure–Phase Transition Behavior Relationships

**DOI:** 10.3390/polym10070794

**Published:** 2018-07-19

**Authors:** Lanying Zhang, Wenhuan Yao, Yanzi Gao, Cuihong Zhang, Huai Yang

**Affiliations:** 1 Department of Materials Science and Engineering, College of Engineering, Peking University, Beijing 100871, China; zhanglanying@pku.edu.cn (L.Z.); gaoyanzi@pku.edu.cn (Y.G.); zhangcuihong@pku.edu.cn (C.Z.); 2 Key Laboratory of Polymer Chemistry and Physics of Ministry of Education, Peking University, Beijing 100871, China; 3 College of Chemistry and Chemical Engineering, Northwest Normal University, Lanzhou 730070, China; wenhuanyao@163.com

**Keywords:** linear and cyclic polysiloxanes, side chain liquid crystal polymers, synthetic strategies, phase structure

## Abstract

Organosilicon polymer materials play an important role in certain applications due to characteristics of much lower glass transition temperatures (*T*_g_), viscosities, surface energy, as well as good mechanical, thermal stabilities, and insulation performance stemming from the higher bond energy and the larger bond angles of the adjacent silicon-oxygen bond. This critical review highlights developments in the synthesis, structure, and phase transition behaviors of polysiloxane-based side chain liquid crystal polymers (PSCLCPs) of linear and cyclic polysiloxanes containing homopolymers and copolymers. Detailed synthetic strategies are elaborated, and the relationship between molecular structures and liquid crystalline phase transition behaviors is systematically discussed, providing theoretical guidance on the molecular design of the materials.

## 1. Introduction

Organosilicon polymers, which can be divided into three categories—polyorganosiloxanes, polyorganosilanes, and polyorganosilicons—according to structural differences in the main chain, have received widespread attention as one of the most important inorganic backbone polymers since commercial products were developed in the 1940s [1,2]. Polyorganosiloxanes (also known as silicones or silicone elasomers) have gained special interest as a result of unique properties such as diverse structures, excellent physical and chemical properties, as well as a wide range of applications [3,4]. From the viewpoint of diverse structures, polyorganosiloxanes can be linear, cyclic, polyhedral oligomeric silsesquioxane (POSS), comb-like, ladder-like, fish-bone like, and so on; the diversity offers infinite space for a variety of performances.

Side Chain Liquid Crystal Polymers (SCLCPs), which integrate the special anisotropy of liquid crystalline materials with the excellent mechanical performance of polymers, have attracted extensive attention over the past few decades [5,6,7,8]. Polysiloxane-based Side Chain Liquid Crystal Polymers (PSCLCPs), distinguishing themselves from traditional polyacrylate-based or polymethacrylate-based SCLCPs with much-lower glass transition temperatures (*T*_g_), viscosities, surface energy, as well as good mechanical, thermal stabilities, and insulation performance stemming from higher bond energy and larger bond angles of the adjacent silicon-oxygen bond [9], have received considerable interest and have been applied in various fields, ranging from liquid crystal display(LCD), information storage, nonlinear optics, gas separation film, etc. [10,11,12], especially areas that require LCPs with a lower phase transition temperature.

PSCLCPs are constructed by attaching the rigid LC mesogens side-on or end-on fixed to the siloxane main chain through flexible spacers. The siloxane main chain can be linear or cyclic, and the rigid LC mesogens can be of any shape (such as rod, disk, or ring) or with different polarities; a tiny change in structure will bring about great changes of materials in nature. Compared with polymers based on carbon-carbon main chain, silicon-oxygen main chain is easier to introduce various LC mesogens into the side chain by post-modification method, making them a promising functional material. Since the first discovery of PSCLCPs by hydrosilylation between hydrogen-containing silicone oil and vinyl liquid crystalline monomers in 1979 by Finkelmann [13], under the guidance of the flexible decoupling strategy [14], a long list of PSCLCPs have been prepared and characterized.

Accordingly, in this critical review, the long list of PSCLCPs is summarized from synthetic strategies and the relationship between the molecular structures and LC phase transition behaviors and structure, in order to provide theoretical guidance for the molecular design of the materials. Influencing factors such as the structures of backbone, species of spacers, etc. are summarized, particularly for the structure–phase transition behavior relationship.

## 2. Synthetic Methods of Polysiloxane-Based Side Chain Liquid Crystal Polymers (PSCLCPs)

From the viewpoint of synthetic methods, the hydrosilylation reaction has dominated for a long time, although there are several other methods, such as esterification, etherification, etc. In recent years, the preparation of a new functional polysiloxane enabled the development of more efficient synthetic methods such as click traction and free radical polymerization, among others. In this section, we will review these reactions in detail.

### 2.1. Hydrosilylation Reaction

**Scheme 1:**
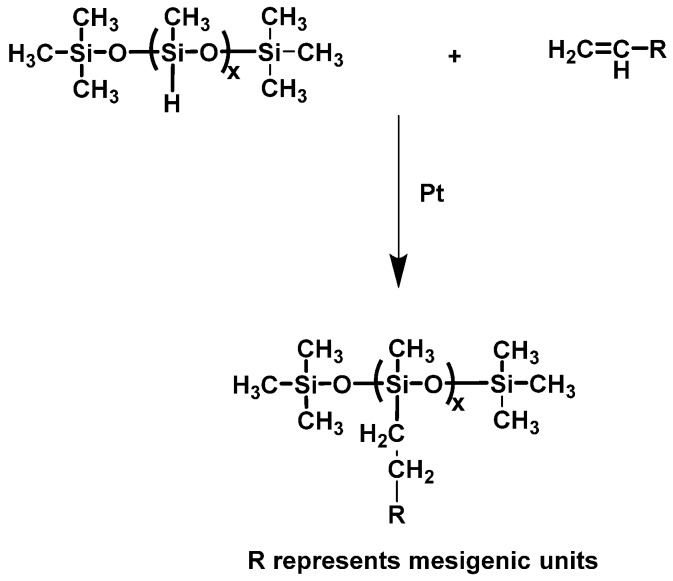
Typical synthetic route of PSCLCPs by hydrosilylation reaction.

Hydrosilylation reaction between polymethylhydrosiloxane (PMHS) and unsaturated carbon–carbon bond based monomers under Pt catalysts was the earliest technique to obtain PSCLCPs, the success of which undoubtedly created conditions for the study of the structure and performance of the organosilicon LCs. Since the first publication of PSCLCPs in 1979 by Finkelmann [13], this method has been a common and most-used method for the synthesis of organosilicon LCs by researchers; this can be ascribed to the ready availability of raw materials, simplicity of the method, as well as easy monitoring of the reaction. A typical synthetic route is shown in Scheme 1.

However, the traditional hydrosilylation method has some imperfections: (1) A small number of unreacted Si–H bond can easily crosslink during post-processing, which is not beneficial for the investigation of the LC phase structure; (2) the coexistence of Markovnikov and anti-Markovnikov addition reactions can result in complexity of the chemical structures and performance of the products, limiting application; (3) difficulty in purification owing to the usage of expensive noble metal platinum catalysts often makes products go grey or black, thus proving unbeneficial for the study of the LC phase structure; (4) because the molecular weight and distribution of hydrogen-containing organic silicon polymers are different, the liquid crystal performance described by different authors may vary greatly, restricting precise referencing. For the above reasons, researches are focusing on improvement measures such as development of platinum/rhodium/palladium complexes with highly efficient catalytic activity, addition of additives, and selection of right solvents, and are also seeking other effective methods [15,16,17,18,19,20].

### 2.2. Chlorination Reaction

**Scheme 2:**
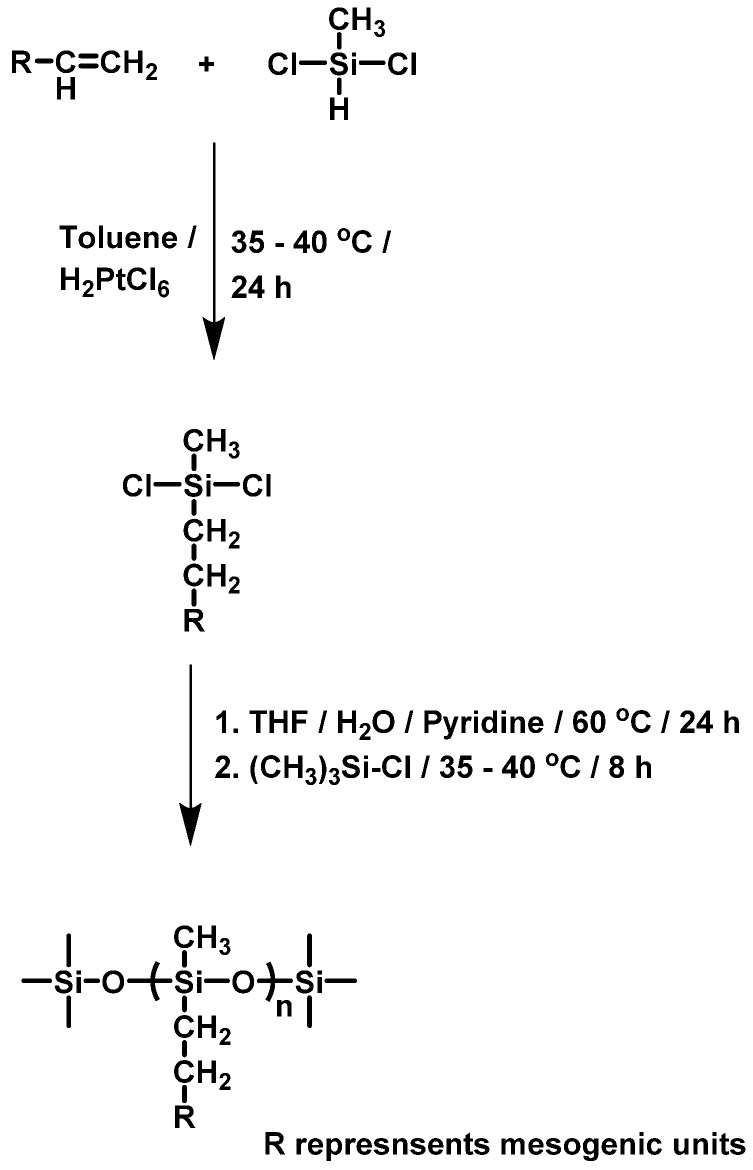
Typical synthetic route of PSCLCPs by chlorination reaction.

Chlorination reaction for PSCLCPs, which means direct polycondensation of the mesogenic dichlolosilanes, is an improvement of the hydrosilylation reaction between polymethylhydrosiloxane (PMHS) and unsaturated carbon–carbon bond based monomers. As shown in Scheme 2 [21], the hydrosilylation reaction between the vinyl mesogens and dichlorosilane was conducted at 35–40 °C in the presence of hexachloroplatinic acid. The polycondensation of mesogenic dichlolosilanes with water was carried out for 24 h; trimethylchlorosilane was then added to produce trimethylsilyl-terminated polymers. The resulting product is purified by repeated precipitation using the THF and methanol system, which results in a notable amount of loss of polymers. However, as an improved method, there are several advantages. It overcomes the incompleteness of the active hydrogen addition reaction, limits easy reduction, solves the difficulty to recycle the Pt catalyst, and restricts generation of crosslinking products stemming from unreacted Si-H bond. This method produces more-ordered PSCLCPs.

### 2.3. Thiol–Ene Click Chemistry

**Scheme 3:**
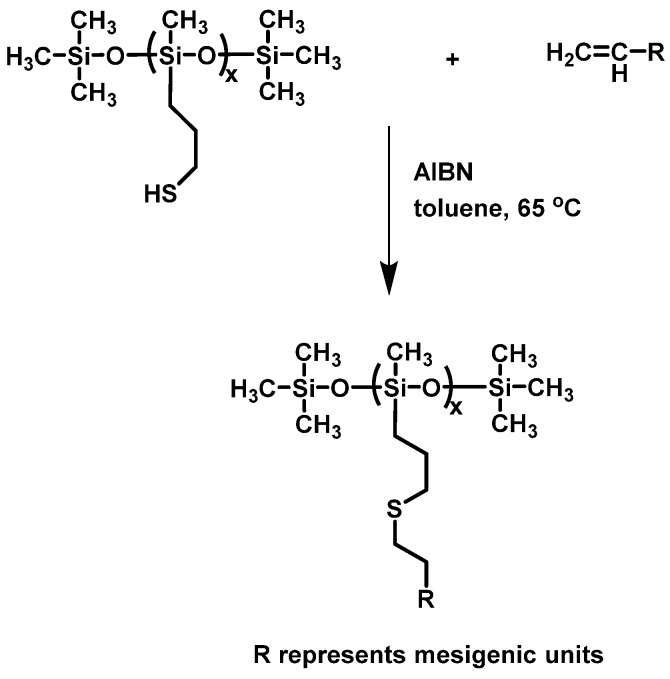
Typical synthetic route and mechanism of PSCLCPs by thiol–ene click chemistry.

Thiol–ene click chemistry, namely hydrothiolation of a C=C bond, as a representative click reaction with high efficiency, strong stereo-selectivity (almost only anti-Markovnikov addition products), simple reaction conditions with no side products, etc., is a very effective method to prepare polysiloxane LCs (the typical reaction route is shown in Scheme 3). Although this method was used in recent years to prepare polysiloxane LCs, it has been well known since 1905 [22]. It has aroused great interest in the development of organic synthetic chemistry, polymer synthetic chemistry, biological chemistry, and materials science. By virtue of the thiol–ene reaction, functional polymer materials with linear [23,24,25], crosslinked [26,27,28], or networked [29,30] structures were designed and synthesized. Reviews on the mechanism of thiol–ene click chemistry and various applications in polymers and materials synthesis were also reasonably elaborated [31,32]. In regard to LC materials, this method has been widely used in the preparation of polymer dispersed liquid crystals (PDLCs) [33,34,35]. H. Yang et al. reported a series of main chain polysiloxane liquid crystal polymers [36,37], side chain polysiloxane liquid crystal polymers [38,39,40], and liquid crystal elastomers (LCEs) [41,42] by thiol–ene click chemistry, in which only anti-Markovnikov addition products were obtained. Although this method overcomes most shortcomings of the hydrosilylation reaction, extensive relevant research has not been carried out due to the relatively few types of raw materials available.

### 2.4. Atom Transfer Radical Polymerization (ATRP) Technique

Atom transfer radical polymerization (ATRP), which represents a simple, inexpensive, and more general method for controlled/living radical polymerization (CRP), was first independently discovered by Mitsuo Sawamoto [43] and by Jin-Shan Wang and Krzysztof Matyjaszewski in 1995 [44,45]. It offers several advantages: tolerance of several functional (such as allyl, amino, epoxy, and hydroxyl) and vinyl groups present in monomers or initiators; ease of preparation; commercially available and inexpensive catalysts (copper complexes); and pyridine-based ligands and initiators (alkyl halides) [46,47]. During the polymerization process, the dormant species is activated by the transition metal complex to generate radicals via the one electron transfer process. Simultaneously the transition metal is oxidized to a higher state. This reversible process rapidly establishes an equilibrium that is predominately shifted to the side with very low radical concentrations. The number of polymer chains is determined by the number of initiators. Each growing chain has the same probability to propagate with monomers to form living/dormant polymer chains (R–Pn–X). As a result, well-defined polymers with controlled molecular weights and narrow molecular weight distribution can be prepared. Accordingly, the control over molecular weight and functionality obtained by ATRP allows for the synthesis of numerous materials with novel topologies, such as graft copolymers [48,49,50,51], star polymers [52,53,54,55], and hyperbranched polymers [56,57,58,59], among others.

**Scheme 4:**
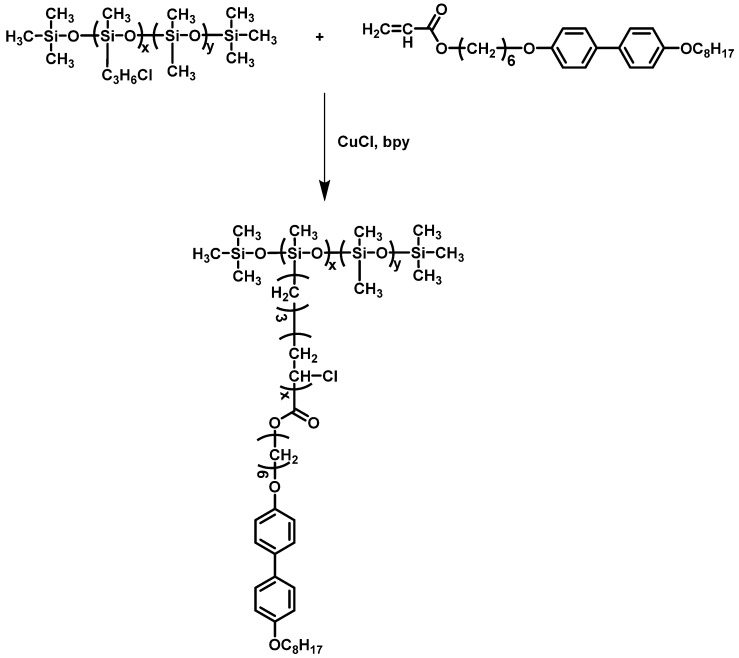
Typical synthetic route of PSCLCPs by atom transfer radical polymerization [60].

Take advantages of ATRP, H. D. Tang et al. first successfully prepared a graft side-chain crystalline polymer PI with polysiloxane as the backbone and a liquid crystal polymer, poly{6-(40-octyloxyphenyl-400-benzoyl)hexyl acrylate}, via atom transfer radical polymerization (ATRP) technique (as shown in Scheme 4) [60]. Differing from conventional polysiloxane LCs, the prepared graft LC polysiloxane contains no Si-H residues, which restricts a subsequent crosslinking reaction by the residual Si–H. Additionally, this method also effectuates the high density of mesogenic units, which have a pronounced effect on the temperature window of the mesophase and the order degree in the mesophase [61,62]. A large number of novel polysiloxane LC materials with complex topologies can be developed using the ATRP method.

### 2.5. Esterification Reaction

Esterification reaction, which can be divided into Fischer Esterification, Steglich Esterification, Yamaguchi esterification, etc., is one of most commonly used methods in organic synthesis chemistry and has been widely used in the manufacture of dyes and medical materials. However, because the reaction is reversible, it does not complete thoroughly.

**Scheme 5:**
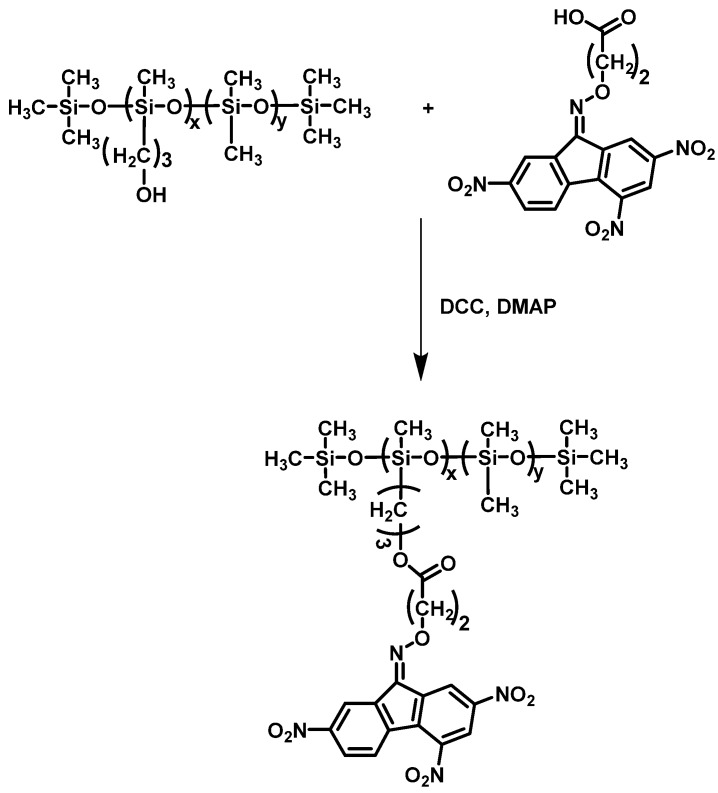
Typical synthetic route of PSCLCPs by Steglich esterification reaction [63].

Utilizing the Steglich esterification reaction, J. H. Wendoff et al. synthesized copolysiloxanes with laterally fixed trinitrofluorenones (TNF) units as side groups (as shown in Scheme 5) [63]. The number-average molar masses of the obtained copolymers are in the range of 3500 g/mol. Due to the limitations of the esterification reaction, which is especially not suitable for the preparation of polymers, this synthetic method is not widely adopted in the synthesis of PSCLCPs.

**Scheme 6:**
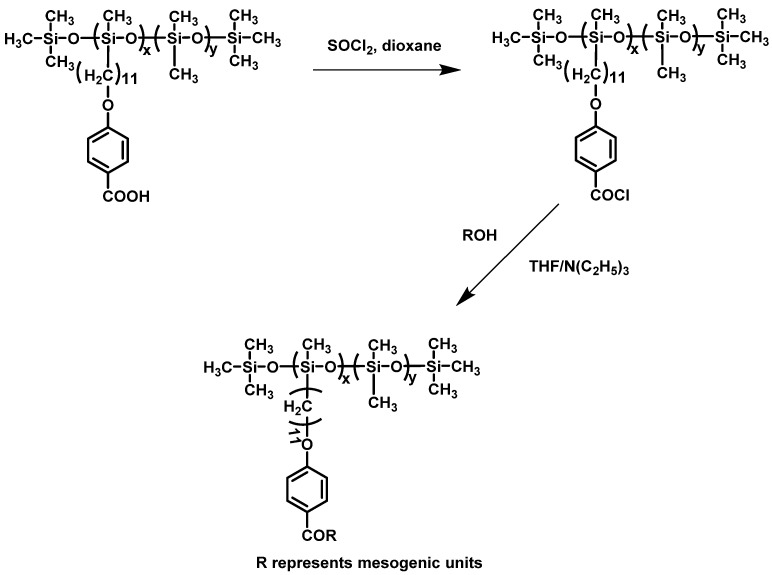
Typical synthetic route of PSCLCPs by acyl chloresterification.

Acyl chloresterification is a mild and efficient method for the synthesis of ester compounds; it can be classified as Steglich Esterification. Accordingly, this reaction can also be used in the synthesis of polysiloxane LCs, which can easily control content and introduce LC mesogenic units. H. D. Tang et al. first synthesized polysiloxanes with carboxyphenyl group in the side chain; then via macromolecule esterification, LC polysiloxanes with side chain of chromophore groups were successfully obtained with a high yield, the structures of which were confirmed by 1H-NMR, IR and elemental analysis (as shown in Scheme 6) [64].

### 2.6. Williamson Nucleophilic Substitution

**Scheme 7:**
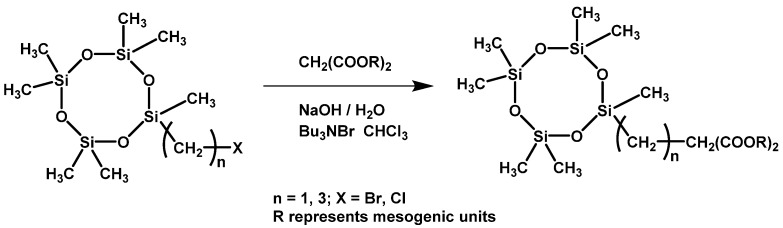
Typical synthetic route of PSCLCPs by Wiliamson nucleophilic substitution.

Williamson nucleophilic substitution is a common method to prepare ether compounds, and is of great significance in organic synthesis. Using this reaction, Zhang X. L. et al. synthesized novel cyclotetrasiloxane materials with mesogens or cyclotetrasiloxane LCs based on halogen-substituted cyclotetrasiloxane under a phase transfer catalyst (as shown in Scheme 7) [65,66]. However, due to the influence of the substrate reactivity, molecular structures and other factors, the Williamson nucleophilic substitution reaction has been found to be suitable for the synthesis of polysiloxane liquid crystal materials with a relative low degree of polymerization.

### 2.7. Supramolecular Interaction

Supramolecular chemistry is the study of entities of greater complexity than individual molecules—assemblies of molecules that bond and organize through intermolecular interactions. The design and synthesis of supramolecular systems invokes interactions beyond the conventional chemical bond, using, for example, hydrogen bonding, Van der Waals forces, dipole-dipole, and π interactions to bring discrete building blocks together. It is of great significance in the field of liquid crystal science to construct liquid crystal and study its properties and applications using supramolecular interactions.

**Scheme 8:**
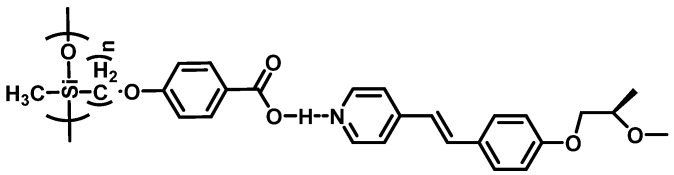
Structure of self-assembled hydrogen-bonded supramolecular PSCLCPs [69,70,71].

Hydrogen-bonded liquid crystalline polymers with well-defined structures, such as main-chain, side-chain, combined, and network structures, have been prepared by the self-assembly of polymers and small molecules. The introduction of non-covalent bonding as a key for the formation of molecularly assembled structures yields a new type of dynamically functional materials. Kato et al. hypothesized proper control of hydrogen bonding, greatly inducing the molecular ordering of thermotropic LCs to obtain a new class of side-chain liquid crystalline polymers [67,68]. Based on the above idea and strategy, Kato et al. synthesized poly(methylsi1oxanes) and poly(methy1-co-dimethylsiloxanes) with side chains containing 4-alkoxybenzoic acid pendant groups attached through aliphatic spacers for use as H-bond donor polymers, and stilbazole derivatives as representative mesogenic or nonmeqogenic H-bond acceptors, respectively. Formation of the liquid crystalline polymeric complexes occurs by self-assembly between the carboxylic acid group of the polysiloxanes with the stilbazoles through H-bonding as confirmed by spectroscopic techniques (as shown in Scheme 8) [69,70,71].

Additionally, ion-counterion interactions may be of importance to liquid crystal ionic polymers [72]. A. Blumstein et al. synthesized a homopolysiloxane with sulfonic ionic site internal and adjacent to the flexible spacer; the interaction of sulfonic ion with pyridine moiety of the small molecule LC resulted in liquid crystalline ionic polysiloxane with quaternized ammonium groups incorporated [73]. B. Y. Zhang et al. also reported the synthesis and characterization of PSLCP ionomers with the sulfonic acid group [74,75]; they were, however, obtained by hydrosilylation reaction of polymethylhydrosiloxane and monomers containing sulfonic acid pendant, which is out of scope of this article.

## 3. The Relationship between the Molecular Structures and LC Phase Transitions & Structures of Polysiloxane-Based Side Chain Liquid Crystal Polymers (PSCLCPs)

Traditional SCLCPs consist of three fundamental parts: the backbone, mesogenic units, and flexible spacers; which determine the generation ability of the LC phase, the LC phase structure type, and phase stability, etc. In this section, we will systematically review the relationship between the molecular structures, and phase transition behaviors and phase structures. To offer more clarity, this section is divided into two parts: The first part discusses the dependence of the type and nature of the main chain, the relative polymerization degree (DP) of the linear polysiloxanes, the ring size of the cyclic polysiloxanes, as well as the shape of the main chain on phase structures and phase behaviors. In the second part, we examine the dependence of the three main spacers (oligooxyethylene, oligomethylene, and thioether) on phase structures and phase behaviors. Special attention is paid to the effect of the heteroatom (such as O, S) in the spacers and their properties. In regard to the dependence of mesogenic units on phase structures and phase behaviors, in addition to the influence of diverse rigid structures [76,77,78]; the lateral substituents of the mesogenic side groups [79,80,81], the aliphatic tail length of the mesogenic groups [82,83,84,85], the chirality [86,87,88,89] and polarity [90] of the mesogenic groups, and the functionality of the mesogenic groups (such as photo-sensitive property [91,92,93,94], amphiphilic property [95,96], etc.) exerted a strong effect on mesomorphic properties. Due to the structural diversity and complexity of mesogenic units, the dependence of mesogenic units on phase behaviors and phase structures of the PSCLCPs is not discussed.

### 3.1. Dependence of the Main Chain on Phase Transition Behaviors & Phase Structures of the PSCLCPs

#### 3.1.1. Dependence of the Type and Nature of the Polysiloxanes Main Chain

Common polymer backbones of SCLCPs are categorized as poly(meth)acrylate, polyvinyl ether, polystyrene, polyacrylamide, polysiloxanes, etc. They can be divided into polyhydrocarbon backbone and polysiloxane backbone, the flexibility and physical properties of which differ. Accordingly, there will be significant differences in the mesophase behaviors.

**Figure 1:**
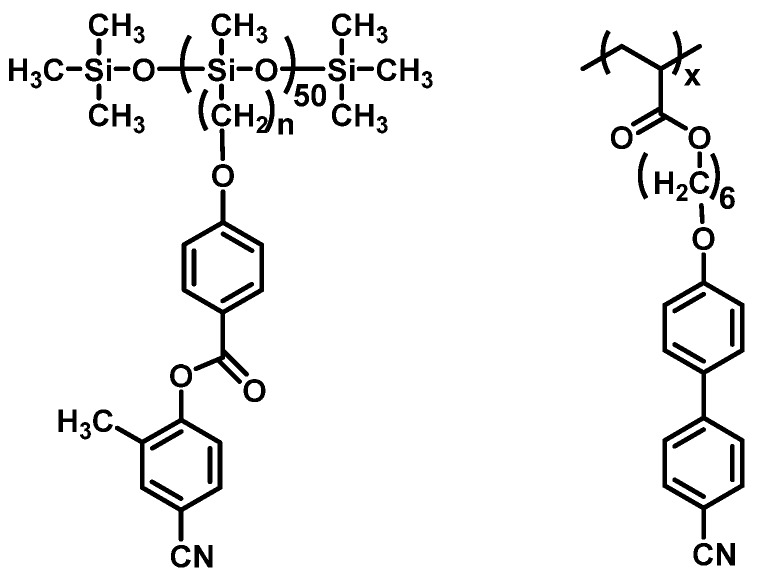
Chemical structures of the polysiloxane and polyacrylate polymers [97].

G. W. Gray FRS, Dr. D. Lacey, and Dr. P. A. Gemmell at Hull University engaged the hydrosilylation reaction to prepare a series of polysiloxanes with 4-cyano-2-methylphenyl benzoate mesogenic unit and different spacer lengths; the chemical structures and their phase transitions data by DSC determination are presented in Figure 1 and Table 1. R. Simon et al. then investigated the dielectric relaxation property of the polymers (discussed in Table 1 [97]). This is evident from a gradual reduction of the activation energy on increasing the temperature for all three polymers in the loss process. The activation energies estimated at a constant reduced temperature (*T* = 0.98*T*_cl_) decrease from 172 and 190 kJ mol^−1^ for nematic polymers with short polymethylene spacers to a value of 95 kJ mol^−1^ for smectic polymer with a longer spacer. In comparison with polyacrylates based on similar structure and polarity of cyanobiphenyl side groups reported by Parneix et al. [98], activation energies ranged from 241 kJ mol^−l^ in a nematic material to 126 kJ mol^−1^ for a smectic polymer; the higher values in evaluation with our results reflect on the fact that the polyacrylate backbone is less flexible than the polysiloxane one. Furthermore, the added relative rigidity of the polyacrylate backbone also generated higher glass transition temperature.

**Figure 2:**
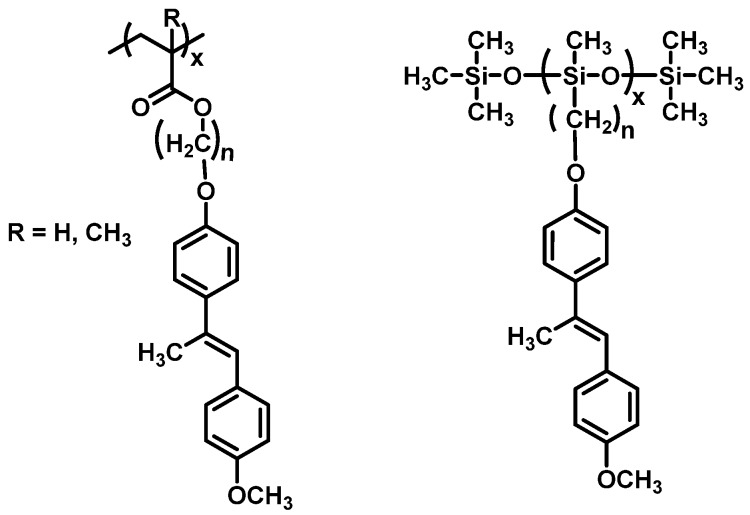
Chemical structures of the Polymethacrylates (4′-n-PMA), Polyacrylates (4′-n-PAC), and Polysiloxanes (4′-n-PS) containing 4-hydroxy-4′-methoxya-methylstilbene based mesogens [99].

V. Percec synthesized polymethacrylates (4′-n-PMA) and polyacrylates (4′-n-PAC) containing 4-hydroxy-4′-methoxy-a-methylstilbene side groups attached to flexible spacers containing 11, 8, 6, 3, and 2 methylenic units and of polysiloxanes (4′-n-PS) containing the same mesogenic group connected through flexible spacers containing 11, 8, 6, and 3 methylenic units, respectively [99]. The chemical structures of the polymers and systematic comparison data are depicted in Figure 2 and Table 2. Results revealed that the nature of the polymer backbone determines the thermal stability of the mesophase and the ease of the side-chain crystallization. That is, for the same spacer length and similar polymer molecular weight, the most-flexible polymer backbone leads to the highest isotropization temperature and the highest ease of side-chain crystallization. This may lead to transformation of enantiotropic mesophases into monotropic mesophases.

**Figure 3:**
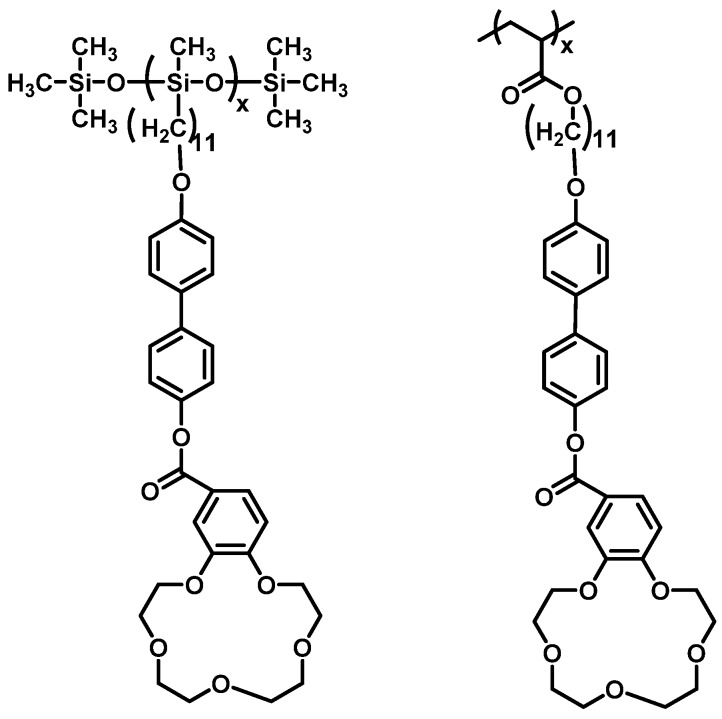
The chemical structures of PSC-11 and PMC-11 [100].

Another work reported by V. Percec was vinyl ether monomers and methacrylate containing benzo-15-crwon-5 groups, by hydrosilylation reaction and conventional free radical polymerization, respectively; the first examples of SCLCPs containing macroheterocyclic ligands within their mesogenic side groups and different backbones were presented (denoted as PSC-11 and PMC-11) [100]. Figure 3 shows the chemical structures of these polymers; the corresponding physical properties are summarized in Table 3. Similar to the results mentioned above, PMC-11 has a polymethacrylate backbone and undergoes isotropization at 166 °C, while PSC-11 with a polysiloxane backbone undergoes isotropization at 216 °C. In addition, PSC-11 displays a second highly ordered smectic mesophase. This last result agrees again with previous results concerning the influence of polymer backbone flexibility on mesomorphic phase transitions [101,102,103].

**Figure 4:**
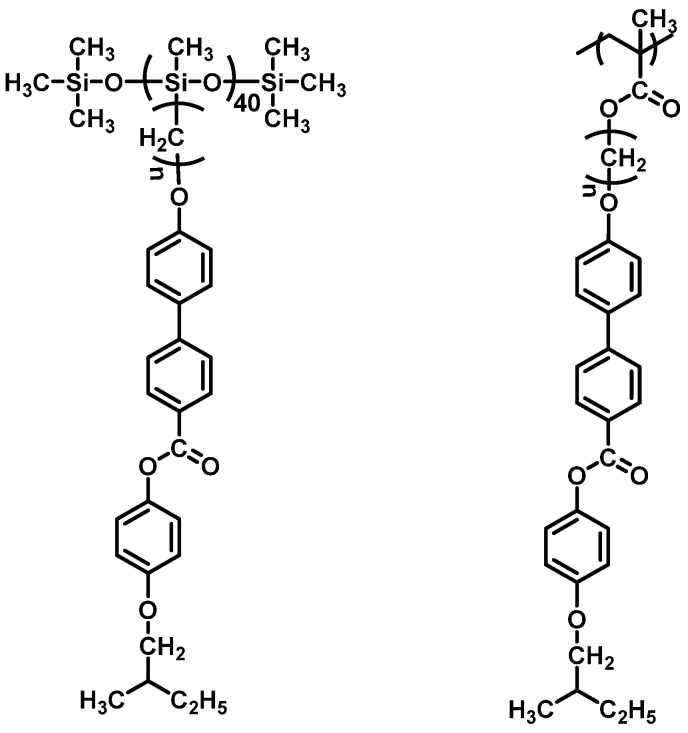
Chemical structures of the polysiloxanes and polymethacrylates [104].

Chain-Shu Hsu et al. designed and synthesized side-chain liquid crystalline polysiloxanes and polymethacrylates that contained 4-[(S)-2-methyl-1-butoxy]phenyl4-hydroxybiphenyl-4′-carboxylate moieties as mesogenic units and 3 to 11 methylene units as aliphatic spacers (the chemical structures of the polymers are shown in Figure 4). Differential scanning calorimetry, optical polarizing microscopy, and X-ray diffraction measurements revealed that flexible polymer backbones enhance the decoupling of the motions of the side chain and main chain and therefore tend to give rise to a higher thermal stability of the mesophases, including the chiral smectic C phase (as summarized in Table 4) [104].

**Figure 5:**
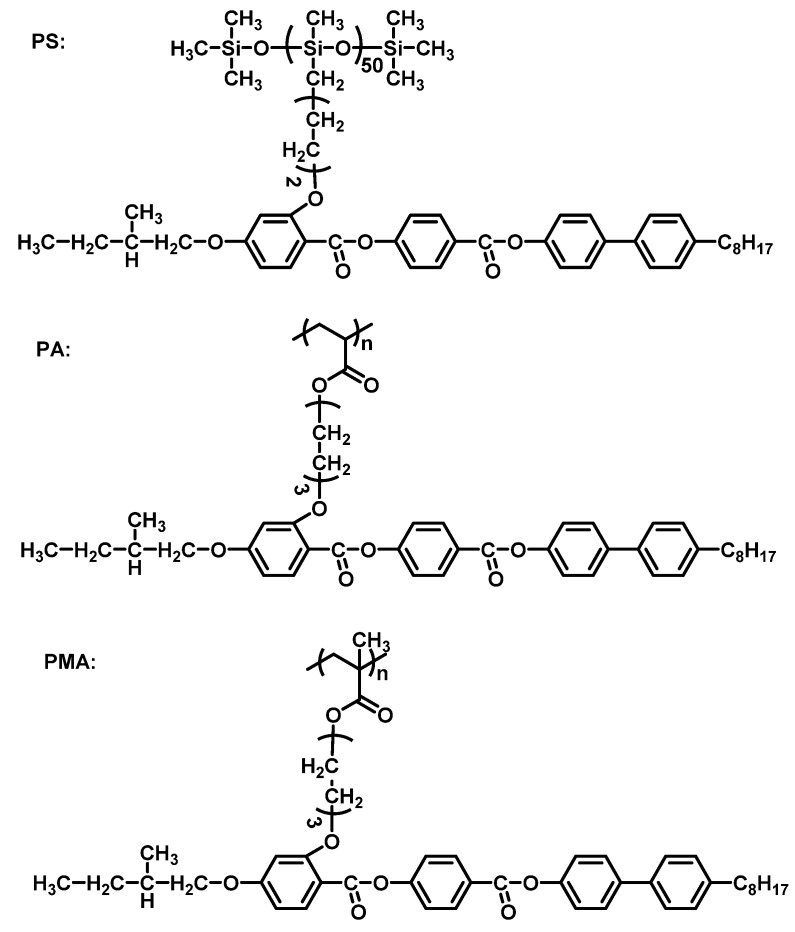
Chemical structures of the polymers based on polysiloxane, polyacrylate and polymethacrylate backbones [105].

Kenneth J. Toyne synthesized a series of chiral, nematic, laterally attached polymers based on polysiloxane, polyacrylate and polymethacrylate backbones, and first showed the comparisons of the effect of different natures of the polymer backbones on phase behaviors (as shown in Figure 5) [105]. Consistent with those reported for polymers with terminally attached side-chains, a decrease in mesophase thermal stability and an increase in glass-transition temperatures were obtained with increase in rigidity of the polymer backbone. Furthermore, the effects of the different polymer backbones in the side-chain systems are more reduced, compared to the related polymers without side-chains (as summarized in Table 5).

Q. F. Zhou synthesized a series of mesogen-jacketed liquid crystalline polymers (MJLCPs) with polysiloxane backbones by hydrosilylation of polymethylhydrosiloxane with styrenic derivatives (as listed in Table 6) [106]. Compared with structurally similar MJLCPs having a polyethylene backbone [107,108,109,110], these MJLCPs based on highly flexible polysiloxane main chain could also self-assemble into supramolecular columnar nematic or smectic liquid crystalline phases; however, the stability of the LC phase was greatly decreased. Contributing to the combined effect of the flexible polysiloxane backbone and the less-rigid side chain structure with the introduction of the ethylene spacer, the glass transition temperatures (*T*_g_’s) of the polysiloxane polymers were about 10 °C lower, and their liquid crystalline ranges were narrowed significantly (as summarized in Table 7).

#### 3.1.2. Dependence of Polymerization Degree of the Linear Polysiloxanes and the Degree of Substitution of the Mesogenic Structural Unit

Generally, phase transition behaviors, LC textures, and stability of the mesophase in MJLCPs greatly depend on molecular weight [107,108,111]. In polysiloxane LC polymers, the influence of molecular weight, composition of mesogenic units, and degree of substitution on LC properties have also been investigated.

**Figure 6:**
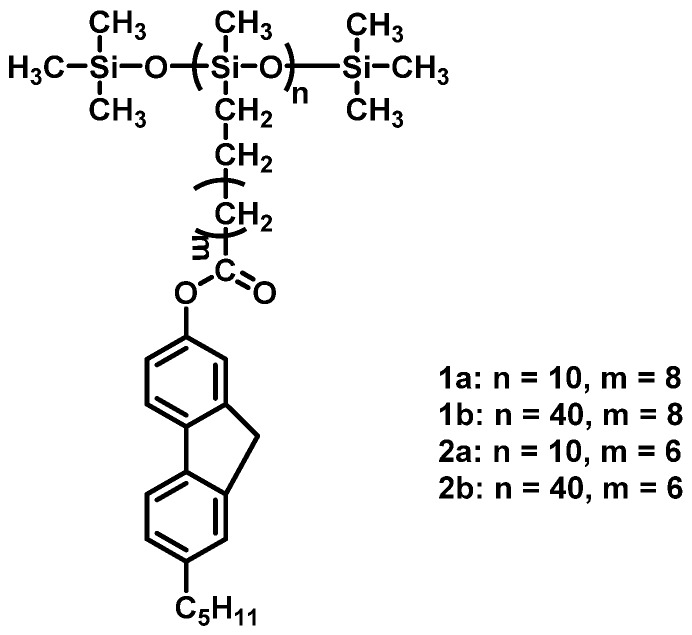
Chemical structures of the polysixanes with fluorene unit [112,113].

G. Kossmehl, et al. synthesized a series of LC polysiloxanes with 2,7-disubstituted fluorene as mesogenic unit and different polymerization degree of the main chain (as shown in Figure 6) [112,113]; the phase behaviour investigation revealed that the longer polymer backbone slightly enhanced the stability of the LC phase, as shown in Table 8.

**Figure 7:**
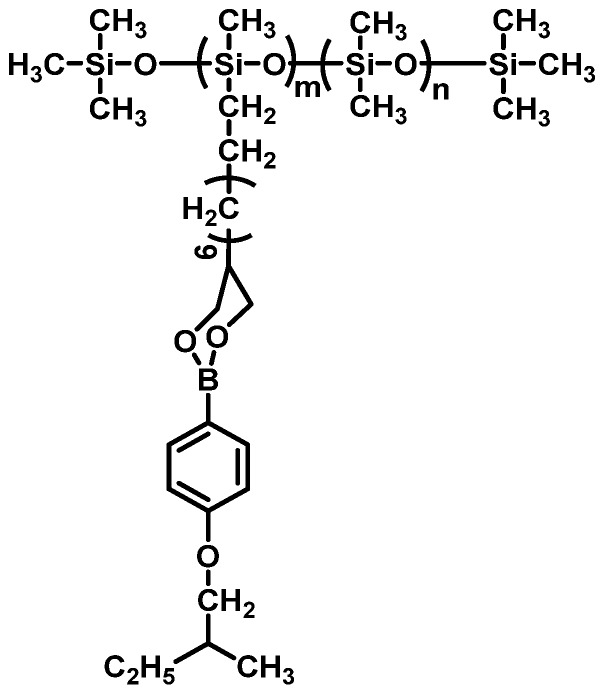
Chemical structures of the polysiloxanes and copolysiloxanes containing mesogenic unit [102].

By the cationic polymerization of 1,3,5,7-tetramethylcyclotetrasiloxane (D4′) and 1,3,5,7-octamethylcyclotetrasiloxan (D4), Percec synthesized a series of poly(methylsiloxane)s and poly(methylsiloxane-co-dimethylsiloxane)s with molecular weight and composition accurately controllable; these can be used as model polymers to study the dependence of molecular weight and substitution degree of mesogenic units on phase behaviors [114,102]. Polysiloxanes and copolysiloxanes containing mesogenic side groups with different molecular weights and compositions were obtained by hydrosilylation reaction between poly(methylsiloxane) and vinyl monomers containing 2-[4-(2(S)-Methyl-1-butoxy)phenyl]-5-(11-undecany1)-1,3,2-dioxaborinane mesogenic unit (as shown in Figure 7). Studies of liquid crystalline properties showed that all the polysiloxanes and copolysiloxanes exhibited a smectic phase, and the phase transition temperatures increased with the polymerization degree (as summarized in Table 9) and composition (as summarized in Table 10) of mesogenic unit. X-ray scattering experiments revealed that the thickness of the smectic layer increased with the structural units containing mesogenic units, and the flexible backbone was squeezed between smectic layers [115,116].

**Figure 8:**
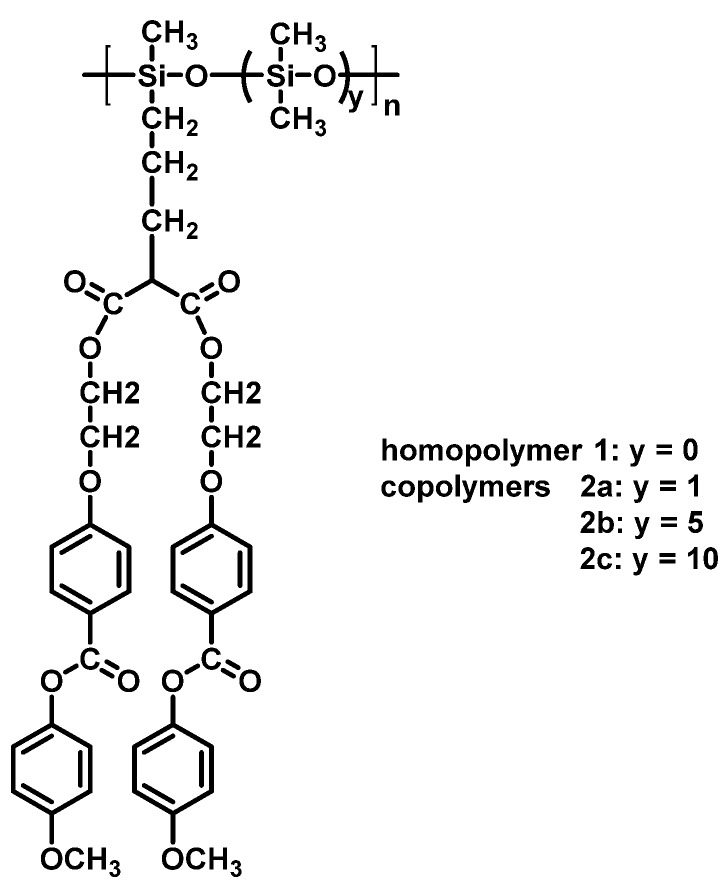
The chemical structures of the liquid crystalline homopolysiloxanes and copolysiloxanes with dimesogenic side groups [118].

**Figure 9:**
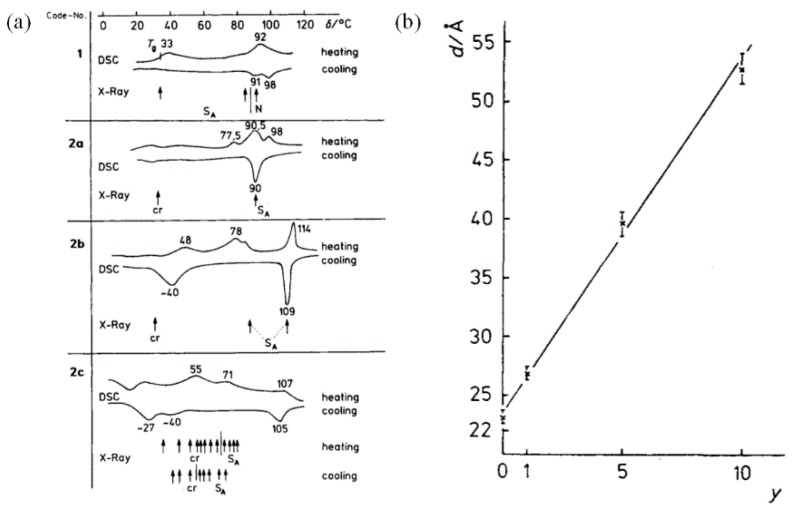
(**a**) Schematic representation of the DSC and X-ray investigation of the homopolysiloxanes and copolysiloxanes (S_A_: smectic A phase, N: nematic phase, I: isotropic phase); (**b**) The layer spacings; (d) as a function of number of the inserted dimethylsioxane unites (*y*) [118].

Diele et al. explored the dependence of the amount of substitution on liquid crystalline structure by means of X-ray diffraction analysis. They synthesized several series of liquid crystalline homopolysiloxanes and copolysiloxanes with different distances between successive dimesogenic side groups linked with the polymer backbone (as shown in Figure 8) [117,118]. Results showed that all the copolymers exhibited smectic phase and two mesophases were found for the homopolymers. The phase transition temperatures of SmA-I increased with the composition of the mesogenic unit. Furthermore, X-ray diffraction results suggested that the smectic monolayer thickness remarkably depended on the amount of substitution, and due to the domaining interaction of the mesogenic groups, a flexible polysiloxane backbone was forced to take part in the layer formation (as illustrated in Figure 9).

**Figure 10:**
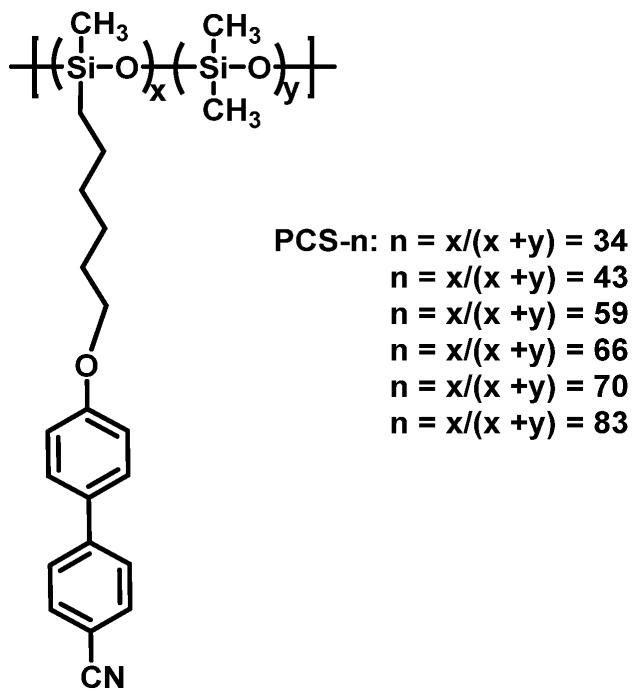
The chemical structures of the poly[6-(4-cyanobiphenyl-4′-oxy)-hexylmetylsiloxanes] (PCS-n) [120].

**Figure 11:**
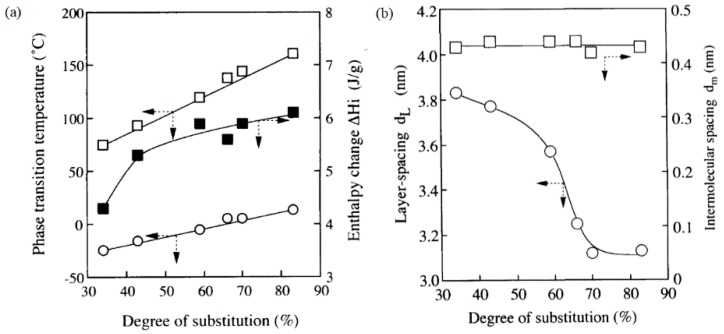
(**a**) Changes in phase transition temperature (*T*_g_ and *T*_i_) and enthalpy change of PCS-n as a function of the degree of substitution (DS); (**b**) Changes in layer-spacing and intermolecular spacing of the PCS-n as a function of the degree of substitution (DS) [120].

Yonetake et al. designed and synthesized poly[6-(4-cyanobiphenyl-4′-oxy)-hexylmetylsiloxanes] (PCS-n, where n denoted the DS, as shown in Figure 10) with various degrees of substitution (DS), and systematically investigated the effects of the degree of the substitution on the phase transition, structures, and the liquid crystallization of PCS-n on the basis of thermal analysis, optical microscope, and X-ray diffraction methods [119,120]. They found that all the PCS-n showed (S_A_) in the temperature range from glass transition temperature (*T*_g_) to clearing temperature (*T*_i_), in spite of various DS. All the *T*_g_ and *T*_i_ increased linearly with DS, and the temperature range of the S_A_ phase between *T*_g_ and *T*_i_ expanded with DS. Since the non-mesogenic flexible backbones were squeezed between smectic between the S_A_ layer, the layer spacing of the S_A_ structure decreased with increasing DS. In addition, due to the entropy relaxation of the backbones between the S_A_ layers, the layer spacing of the sample with lower DS highly decreased with increasing temperature. Furthermore, the liquid crystallization of PCS-n was remarkably influenced by DS; PCS-n with higher DS exhibited faster growth rates of liquid crystalline textures compared with lower DS (as shown in Figure 11).

**Figure 12:**
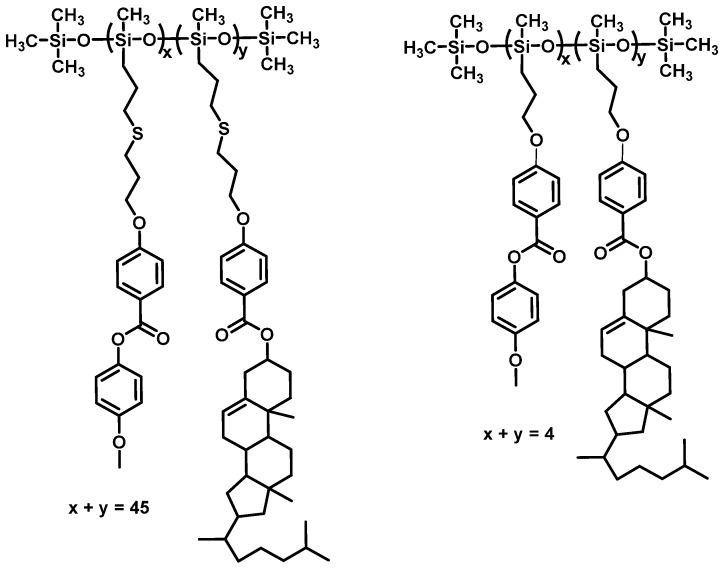
Chemical structures of the copolymers based on PMMS backbone and linear siloxane tetramer backbone [121,122].

H. Yang et al. synthesized a series of PSLCPs copolymers based on poly[3-mercaptopropylmethylsiloxane] (PMMS) (M.W. 4000–7000) with chiral cholesteric-based group and achiral benzene methyl ether group via thiol–ene click chemistry. A battery of linear siloxane tetramers containing the same chiral and achiral mesogenic groups were also obtained by traditional hydrosilylation reaction (the chemical structure of the polymers is shown in Figure 12) [121,122]. Contributing to the increased length of the polysiloxane backbone and spacer as well as the cooperative interaction between them, a more ordered LC phase of smectic E (S_m_E) developed spontaneously at low temperatures for polymers with chiral cholesteric unit (*X*_chol_) below 0.3 and a strong dependence of LC phase structures on the molar content of the chiral group was observed. However, for the linear siloxane tetramers, a lower degree of polymerization generated only cholesteric or smectic A phase with rather wide temperature ranges (see Table 11).

#### 3.1.3. Dependence of the Ring Size of the Cyclic Polysiloxanes Main Chain

**Figure 13:**
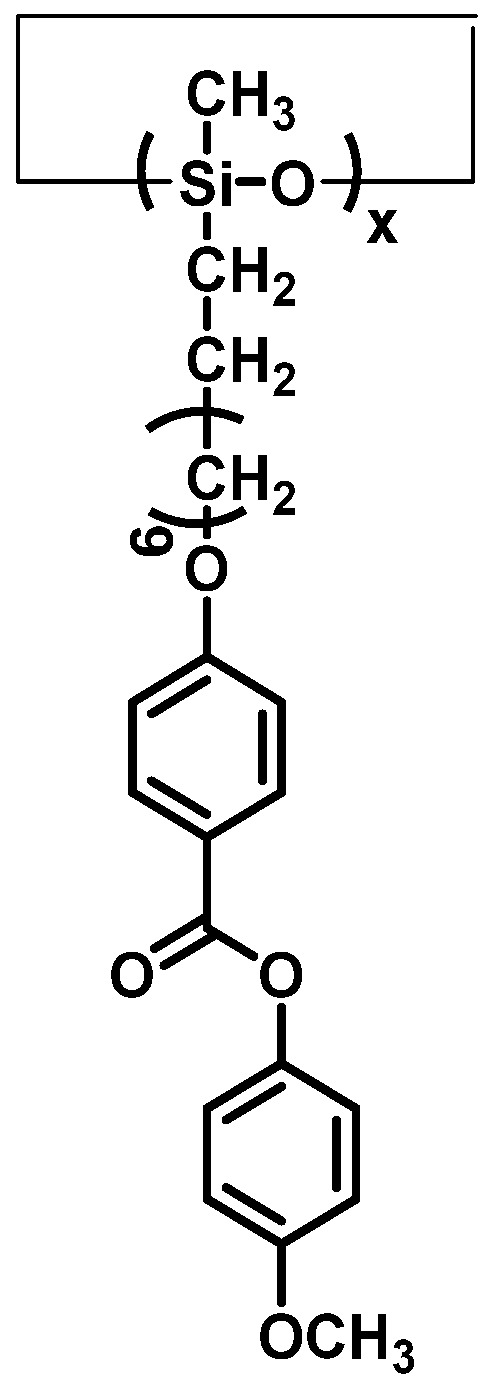
Chemical structures of the liquid crystalline oligo[methyl(hydrogen)cyclosiloxanes] [123].

The ring size of the cyclic compounds determines its molecular rigidity, which strongly influences the phase behaviors. For liquid crystalline oligo(cyclosiloxanes), the effect is even more pronounced. W. Staficzyk synthesized a series of well-defined liquid crystalline oligo[methyl(hydrogen)cyclosiloxanes (the molecular structures are shown in Figure 13) [123]. Differing from the work by Gray et al. [124], W. Staficzyk found a close relationship between the size of the ring and the crystal-smectic phase transition temperature (as shown in Table 12). With increase of the ring size of oligo(cyclosiloxanes), a decrease in the temperature from 50 °C to 38–37 °C is directly correlated with an increase in d value from 3.13 nm for D4 to 3.27 nm for D8, which may probably be due to the more conformational freedom of larger rings. 

**Figure 14:**
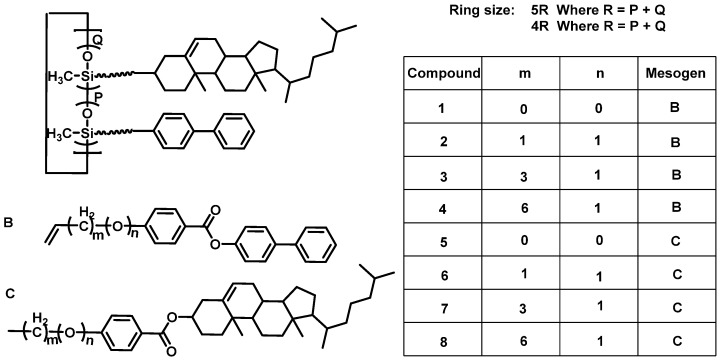
The chemical structures of the cyclic siloxane LCPs [125].

By conventional hydrosilylation chemistry, T. J. Bunning et al. synthesized a series of cyclic siloxane LCPs based on pentamethylhydrosiloxane and tetramethylhydrosiloxane rings as well as mesogens with cholesterol and biphenyl [125]. The chemical structures are listed in Figure 14; the variables examined included ring size, spacer group length, and type and composition of pendant mesogenic groups. Results revealed that the influence of the ring size on the phase behaviors has to do with the mesogenic unit and spacers length. For homopolymers and copolymers with more rigid mesogenic unit and shorter spacer length, greater impact on phase behaviors can be found. But overall, the tetramethyl ring derivatives exhibited higher glass transition or crystallization temperatures than their pentamethyl counterparts due to more rigidity of the tetramethyl ring (As illustrated in Table 13).

#### 3.1.4. Dependence of the Shape of the Polysiloxanes Main Chain

The shapes of the polymer chain (e.g., linear, cyclic, branched, or networked), which strongly influence the flexibility and tacticity of the polymer, have a pronounced effect on the phase behaviors of the SCLCPs. Polysiloxanes have diverse backbone structures, such as linear and cyclic polysiloxane [126,127], tetrahedral or octathedral silsesquioxane [128,129], branched or networked polysiloxane [130,131], etc., the influence of which on LC properties have also been investigated.

**Figure 15:**
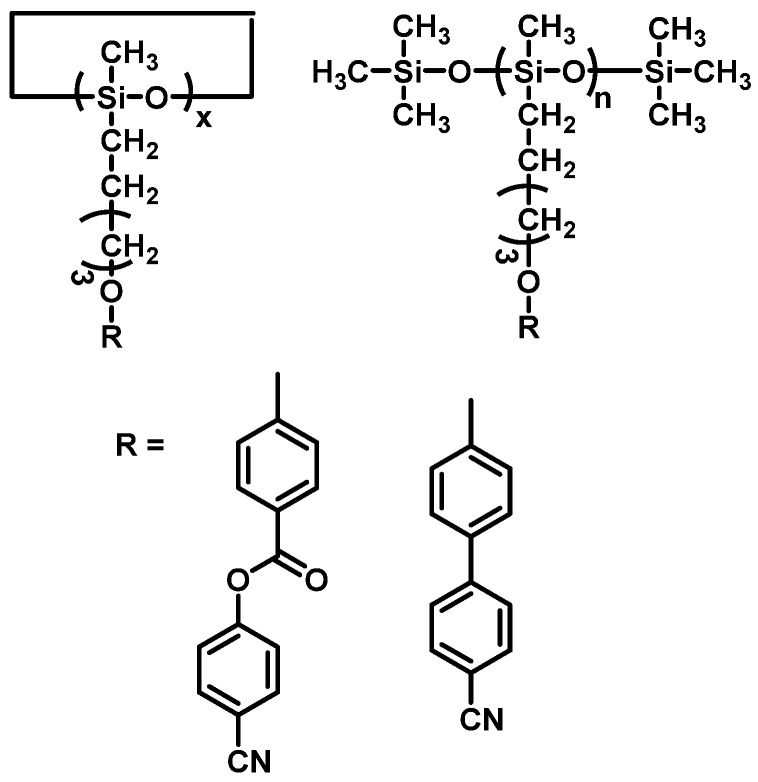
The chemical structures of the cyclic and linear polysiloxanes [124].

**Figure 16:**
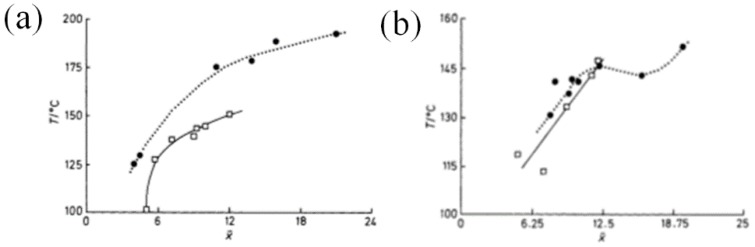
(**a**) Mesophase-isotropic transition temperatures for cyclic (•) and linear (▪) polysiloxanes containing the cyanoester mesogenic groups; (**b**) Mesophase-isotropic transition temperatures for cyclic (•) and linear (▪) polysiloxanes containing the cyanobiphenyl mesogenic groups [124].

G. W. Gray et al. synthesized a series of cyclic SCLC polysiloxane and their open chain analogues by means of a standard hydrosilylation reaction using both cyclic (the number-average degree of polymerization were approximately between 4 and 24) and linear (the number-average degree of polymerization were approximately between 4 and 13) polymers [124]. The chemical structures of the polysiloxanes are shown in Figure 15. The results reveal that the mesophase-isotropic transition temperatures of the cyclic polymers were generally higher than those of the linear analogues (Figure 16a,b), which may be attributed in part to the absence of the trimethylsilyl end groups in the cyclics. Such end groups would be expected to dilute the mesophase, thereby lowering the transition temperatures of the linear relative to those of the corresponding cyclic materials.

**Figure 17:**
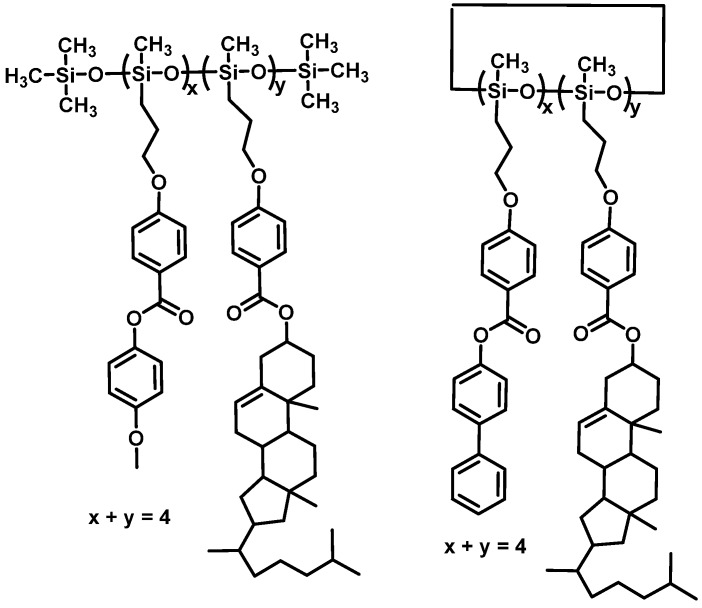
Chemical structures of the cyclic and linear polysiloxane tetramers [122,132].

H. Yang et al. designed and synthesized cyclic and linear polysiloxane tetramers with similar mesogenic groups and investigated the influence of the shape of tetramer backbone on the phase behaviors [122,132]. The chemical structures of the polysiloxanes are shown in Figure 17. The results reveal that owing to the increased rigidity of the cyclic backbone, abundant LC phase structures with blue phase (BP) and relatively high glass transition temperatures were discovered (see Table 14). They pointed out that the increased rigidity of the cyclic backbone played an important role in the stability of the blue phase, which accordingly improved the helical twisting power (HTP).

### 3.2. Dependence of the Flexible Spacer on Phase Transition Behaviors & Phase Structures of the PSCLCPs

The flexible spacers between the mesogenic side group from the polymer backbone, which can usually improve the mobility of the mesogenic groups and facilitate the formation of LC phase structures, plays an important role in determining many physical properties of the resulting liquid-crystal polymers [133,134,135]. According to the flexible decoupled theory, the spacers can provide an effective decoupling between the polymeric chain and the mesogenic groups. Generally, the longer the spacer is, the more stable the mesophase is. From the perspective of application, elasticity is a very important factor; the longer the spacer, the lower the elasticity constant is. Accordingly, increase in flexibility facilitates response to the electric or magnetic fields.

For PSCLCPs, there are mainly three kinds of spacers: polymethylenic, polyoxyethylenic, and thioether. Due to differences in size and nature of carbon, oxygen, and sulfur atoms, the physical properties of the corresponding polymers are obviously different, and interesting applications may be made. For example, polymethylenic flexibility of the spacer is significantly increased by the presence of oxygen and sulfur, which bring about a difference in the sensitivity to electric, optical, magnetic fields.

#### 3.2.1. Dependence of the Shape of the Polysiloxanes Main Chain

For all the linking spacers, the polymethylenic spacer is the most commonly used because it is easily carried out and offers good results. The longer the polymethylenic spacer, the more stable the mesophase is. Furthermore, increase of the polymethylenic spacer enables the development of more abundant and ordered LC structures such as S_C_, S_E_, etc. [136,137,138,139]. However, crystallization often occurs in some early reported PSCLCPs, which is a disadvantage for application. Several research works were carried out to identify ways to decrease or avoid this phenomenon. Percec et al. introduced structural or conformational isomer mesogenic groups in copolymers to remove the crystallization [140,141,142]. Similarly, Chain S. HSU et al. synthesized polysiloxanes homopolymers with cyclohexane based mesogenic units, which exhibited conformational isomerism; the side chain crystallization was efficiently avoided, even with usage of very long spacers [143]. In addition, crystallization can also be limited by carrying out block copolymers possessing mesogen structures with non-mesogen ones [144], or by introduction of a voluminous group in spacers [145].

#### 3.2.2. Dependence of the Polyoxyethylenic Spacer on the Phase Transition Behaviors & Phase Structures of the PSCLCPs

Oligo(oxyethylene)s groups, which exhibit polarity, flexibility, hydrophilicity, low toxicity, and the ability to form complexation with Li^+^, have an important position in the field of physical chemistry and other scientific research, and have been used in molecular engineering of rigid rod-like conjugated polymer systems in order to improve solubility in hydrophilic solvents [146], or to obtain “hairy rod” polymers for polymer electrolyte applications [147,148,149].

**Figure 18:**
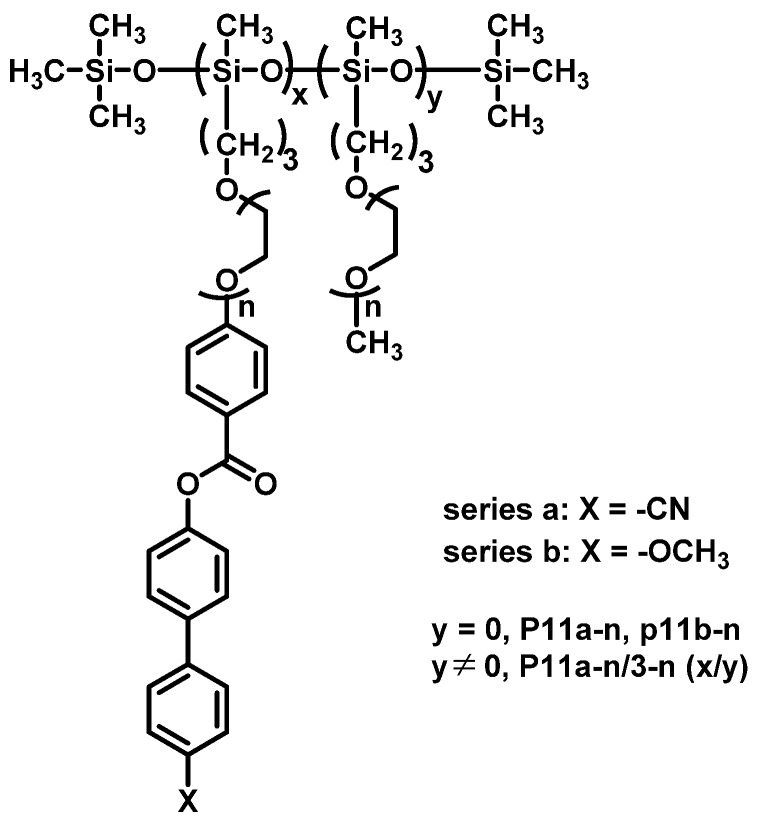
Chemical structures of the side chain LC homo-polysiloxanes and co-polysiloxanes containing end-on fixed mesogens and oligooxyethylenic spaces [150].

**Figure 19:**
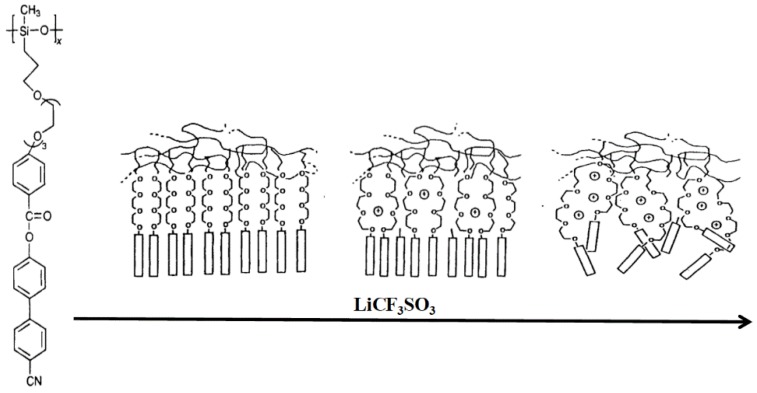
Schematic representation of the mechanism of salt complexation and its effect on the arrangement of the mesogenic side groups of polysiloxanes containing oligooxyethylenic spacers [150].

Percec et al. designed and synthesized side chain LC homo-polysiloxanes and co-polysiloxanes containing end-on fixed mesogens and oligooxyethylenic spaces (see Figure 18), and systematically investigated the effects of length of oligooxyethylenic spacers and complexation with LiCF_3_SO_3_ on their thermotropic mesomorphic behaviors [150]. As shown in Table 15, with the increase of the spacer length, the glass-transition temperatures of the homo-polysiloxanes and co-polysiloxanes increased, while the smectic-isotropic (S-I) transition temperatures decreased. Similarly, (in Table 16), the complexation of the polysiloxane based on the monomer lla-3 (i.e., P-lla-3) with LiCF_3_SO_3_ also led to an increase in their glass-transition temperatures and a decrease in their S-I transition temperatures. These complexes are thermotropic liquid-crystalline polyelectrolytes, which can accommodate up to 0.6 mol of salt per mole of ethylene oxide repeat unit (CH_2_CH_2_O). Figure 19 presents the possible mechanism of the complexation of LiCF_3_SO_3_ by the oligooxyethylene spacer and its effect on the arrangement of the mesogenic units of the polymer in the S_A_ mesophase. At low LiCF_3_SO_3_ per mru ratios, the complexation of LiCF_3_SO_3_ results in a stiffening of the side chains and consequently in a decrease in segmental mobility; *T*_g_, therefore, increases. On the other hand, the flexibility of the spacer decreases and its conformation changes. Therefore, the SA-I transition temperature decreases. When the amount of LiCF_3_SO_3_ becomes high enough so that the majority of the oligooxyethylene of the spacer are participating in complexation, a distortion in the arrangement of the mesogenic side-groups should occur. Consequently, the formation of the S_A_ mesophase cannot be completed.

**Figure 20:**
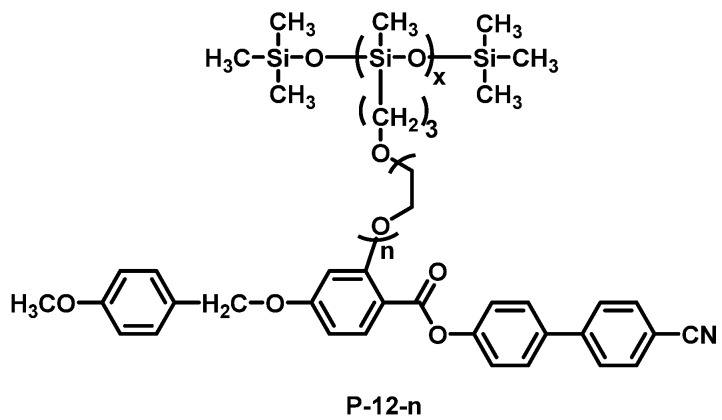
Chemical structures of the side chain LC homo-polysiloxanes containing side-on fixed mesogens and oligooxyethylenic spaces [151].

**Figure 21:**
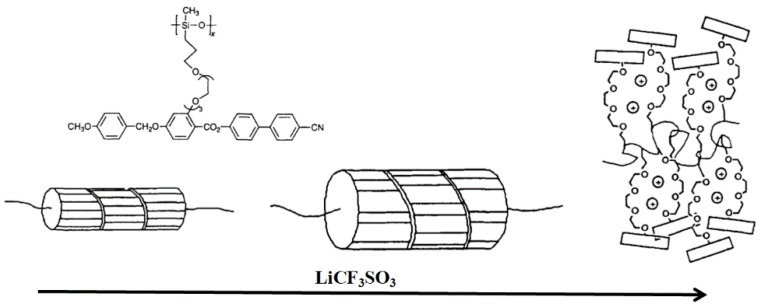
Schematic representation of the mechanism of salt complexation and its effect on the arrangement of the mesogenic groups of a side chain LC homo-polysiloxanes based on oligooxyethylenic spacers and side-on fixed mesogenic groups [151].

Percec et al. also designed and synthesized the first examples of side chain LC homo-polysiloxanes containing oligooxyethylenic spacers and side-on fixed mesogenic groups (see Figure 20), and investigated systematically the effect of oligooxyethylenic spacers on their phase transition behaviors [151]. Results revealed that all the polysiloxanes synthesized displayed enantiotropic N mesophases, and *T*_g_ and *T*_i_ decreased obviously with the length of spacers (see Table 17). The complexation of the polysiloxane P-12-3 with LiCF_3_SO_3_ resulted in an initial decrease and then a subsequent increase of the glass transition of the complexes, and in a decrease of the N-I transition temperature (as shown in Table 18). These complexes are nematic liquid-crystalline polyelectrolytes up to a value of 0.4 mol LiCF_3_SO_3_ per mole repeat unit of polymer. Above this value, the N mesophase cannot form owing to its close proximity to the glass-transition temperature. A possible explanation for this behavior can arise from the consideration of the mesogen model previously described [152]. According to this model, the helical arrangement of the mesogenic units is responsible for the formation of the N liquid-crystalline phase. A schematic representation of the helical arrangement of the mesogenic units of the uncomplexed polymer in the N mesophase is shown in Figure 21. Complexation with LiCF_3_SO_3_ results in incorporation of Li^+^ ions within the cylindrical structure and, consequently, to an increase in its diameter. Eventually, increased LiCF_3_SO_3_ complexation destroys the helical arrangement and transforms it into an open structure in which the nematic arrangement of the mesogens is highly distorted, leading to the disappearance of the liquid-crystalline mesophase.

#### 3.2.3. Dependence of the Thioether Spacer on the Phase Transition Behaviors & Phase Structures of the PSCLCPs

In the periodic table of chemical elements, sulfur atoms and oxygen atoms belong to the same family. However, the larger atomic radius of sulfur atoms results in a significant difference in polarity and compliance with oxygen atoms, which may lead to a more sensitive response to electro-optical performance.

**Figure 22:**
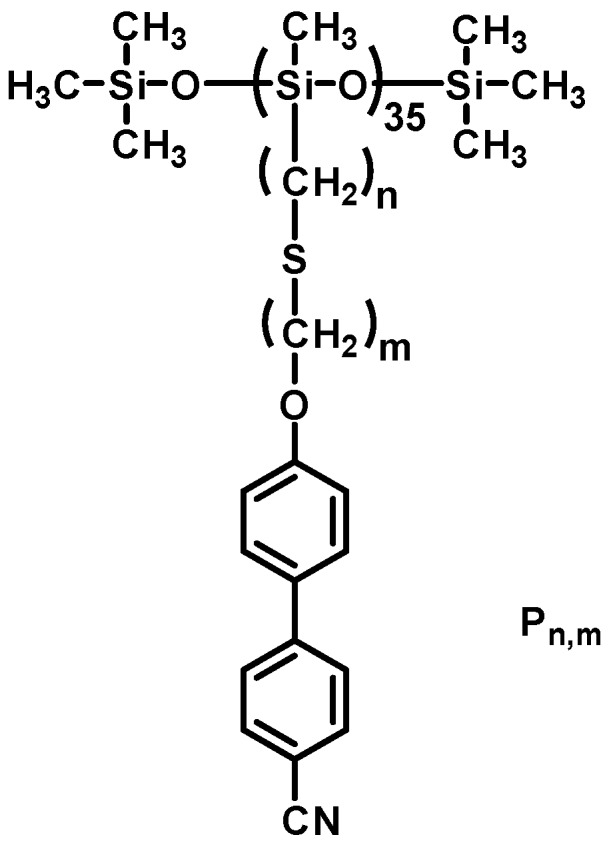
Chemical structures of the cyanobiphenyl-based polysiloxanes containing long thioether spacers [154].

**Figure 23:**
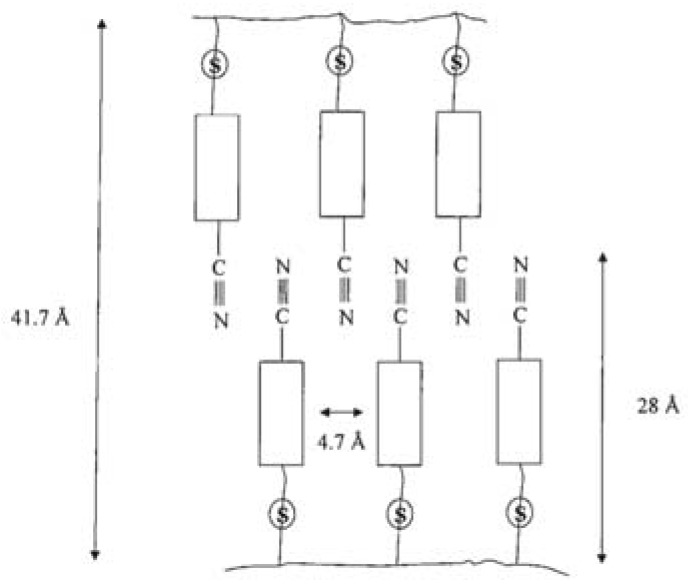
Example of an “interdigitated” structure observed by X-ray analysis: P8, 2 [154].

Jean-Claude Milano et al. developed three new cyanobiphenyl-based polysiloxanes containing long thioether spacer [153,154]; Figure 22 presents the chemical structures. This type of spacer effectively removes the crystallinity phenomenon, enables to obtain a wide range of mesophase and to get polymers presenting an interdigitated smectic A (S_m_A_d_) phase structure at room temperature (as shown in Table 19 and Figure 23). Compared with the polymethylenic spacer, the flexibility of the thioether spacer is increased by the presence of sulfur, which could have interesting consequences on the properties of these polymers, e.g., the sensitivity to electric, optical, magnetic fields.

**Figure 24:**
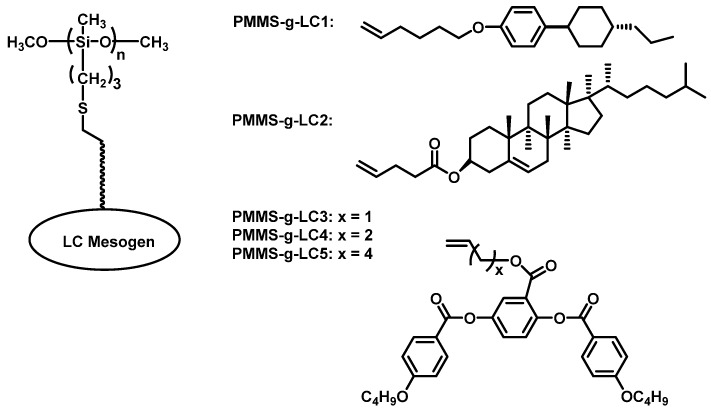
Chemical structures of the polysiloxane-based LCPs with thioether spacer via thiol−ene click chemistry [39].

H. Yang et al. developed a series of polysiloxane-based LCPs with thioether spacer via thiol−ene click chemistry (as shown in Figure 24) [39]. On the basis of the POM, DSC, SAXS, and WAXS results, these polysiloxane-based LCPs with thioether spacer could readily achieve a wide variety of liquid crystalline phases of nematic (N) phase or smectic (S_m_X) phase. Due to the lack 2D WAXD results, the precise smectic structure of PMMS-g-LC1 and PMMS-g-LC1 was not confirmed. However, according to Dreiding models [155,156] and the results obtained by Jean-Claude Milano [153,154], PMMS-g-LC1 and PMMS-g-LC2 might have S_m_A and interdigital S_m_A_d_ phases, respectively (as illustrated in Table 20). 

**Figure 25:**
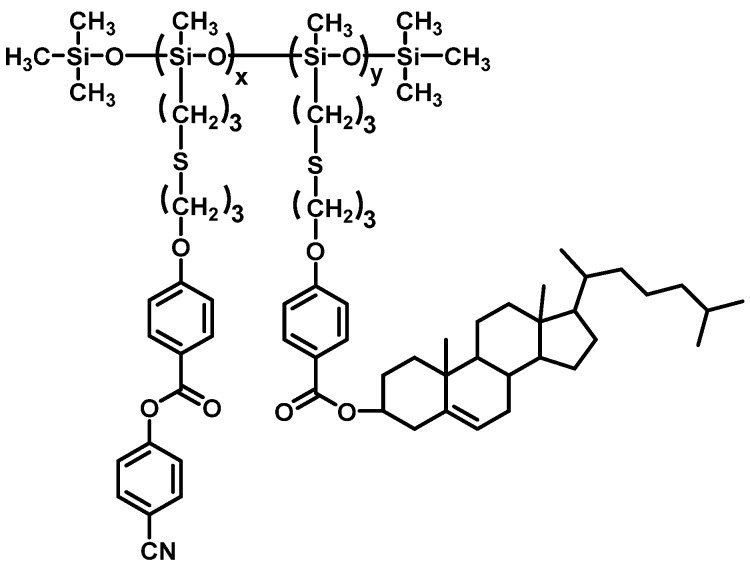
Chemical structures of PMMS-CN-based polymers [90].

By thiol-ene click chemistry, H. Yang et al. designed and synthesized a series of PMMS-based PSCLCPs, the mesogenic units of which were cholesterol-terminated chiral unit with achiral substituted 4-methoxyphenyl 4-(allyloxy) benzoate unit [121], or cholesterol-terminated chiral unit with achiral substituted 4-cyanophenyl 4-(allyloxy) benzoate unit [90], respectively. The phase behaviors of PMMS-based polymers based on cholesterol-terminated chiral unit and achiral substituted 4-methoxyphenyl 4-(allyloxy) benzoate unit (abbreviation for PMMS-OCH_3_-based polymers) have been described in this paper above. However, the polymers based on cholesterol-terminated chiral unit with achiral substituted 4-cyanophenyl 4-(allyloxy) benzoate unit (abbreviation for PMMS-CN-based polymers, the chemical structure of which is shown in Figure 25) exhibited a drastic change in mesogenic state although only cyano substitution was used instead of methoxy substitution. Attributing to the decisive role of the polarity interaction, a strong dependence of LC phase structures on *X*_chol_ did not reappear in this series of cyano-terminated polymers. With increasing *X*_chol_, polymers with cyano-terminated side chains in this work developed a single monolayer interdigitated S_m_A phase (as illustrated in Table 21). The results obtained above were all in accordance with those by Jean-Claude Milano [153,154].

## 4. Conclusions

Encouraged by the first publication of PSCLCPs via hydrosilylation reaction between polymethylhydrosiloxane (PMHS) and unsaturated carbon–carbon bond based monomers, a variety of improved synthetic methods and lots of polysiloxane-based LC materials with diverse structures were rapidly developed. We attempted to find the factors influencing phase transition behaviors and phase structures from a molecular level. The following trends can be concluded: For the influence factors of main chain, (i) flexible polysiloxane backbones that can enhance the decoupling of the motions of the side chain and main chain tend to give rise to a higher thermal stability of the mesophases, and a decrease in glass-transition temperatures. However, for cyclic SCLC polysiloxane and corresponding open chain analogues, increased rigidity of the cyclic backbone played an important role in the stability of the blue phase, which accordingly improved the helical twisting power (HTP); (ii) A longer polysiloxane backbone (namely, a higher degree of polymerization, DS) can enhance the stability of the LC phase, and even more ordered phase structures can be developed; (iii) The phase transition temperatures increased with the polymerization degree (DS) and composition of the mesogenic unit. All the *T*_g_ and *T*_i_ increased linearly with DS, and the temperature range of the LC phase between *T*_g_ and *T*_i_ expanded with DS. For the influence factors of spacer, (i) The flexible spacers between the mesogenic side group from the polymer backbone can usually improve the mobility of the mesogenic groups and facilitate the formation of LC phase structures. Generally, the longer the spacer is, the more stable the mesophase is; (ii) The increase in rigidity and polarity of the spacer can increase the glass transition of the polymers, and widen the range of the mesophase. However, too much increase in rigidity of the spacer may also destroy the ordered arrangement of the molecules, and lead to disappearance of the liquid-crystalline mesophase; (iii) The introduction of heteroatoms such as oxygen and sulfur atoms in the spacer can properly improve the flexibility and polarity of molecules, which could have interesting consequences on the properties of these polymers, e.g., the sensitivity to electric, optical, and magnetic fields. The analysis above will help us design molecular structures effectively.

## Figures and Tables

**Table 1:** The phase transition and activation energies of polysiloxanes and polyacrylate polymer [97,98].

**Polymer**	**Spacer Length**	**Phase Transitions (°C)**	**Activation Energies (kJ mol^−1^)**			
			**At Low Temperatures in LC Phase**	**At 0.98*T*_cl_**		**Isotropic Phase**
polysiloxane	5	G 19 N 65 I	202	172		142
polysiloxane	6	G 7 N 48 I	238	190		144
polysiloxane	8	G 9 S 102 I	146	95		––
**Polymer**	**Spacer Length**	**Phase Transitions (°C)**	**Activation Energies (kJ mol^−1^)**			
			**N_Re_**	**S_Ad_**	**N**	**I**
polyacrylate	6	G 32 N_Re_ 80 S_Ad_ 124.5 N 132 I	241	126	193	83

Note: G: glass state; N: nematic phase; S: smectic A phase; I: isotropic phase; *T*_cl_: temperature at the liquid crystalline-isotropic phase transition; N_Re_: re-entrant nematic phase; S_Ad_: smectic A phase with a double layer.

**Table 2:** Thermal transition and thermodynamic parameters of polymethacrylates (4′-n-PMA), polyacrylates (4′-n-PAC), and polysiloxanes (4′-n-PS) [99].

Polymethacrylates (4′-n-PMA)	GPC		Phase Transitions(°C) and Corresponding Enthalpy Changes (kcal/mru^−1^)	
	10^−3^$\bar{M}$_n_	$\bar{M}$_w_/$\bar{M}$_n_	Heating	Cooling
4′-11-PMA	23.4	2.0	G 12 S 116 (1.25) I	I 105 (1.13) S 4 G
4′-11-PMA ^a^	23.4	2.0	G 15 K 55 (0.55) K 83 (0.39) S 119 (0.99) I	I 105 (1.13) S 4 G
4′-8-PMA	24.1	2.0	G 18 S 55 (0.29) N 112 (0.43) I	I 108 (0.43) N 53 (0.21) S 15 G
4′-6-PMA	24.7	2.5	G 32 N 101 (0.26) I	I 97 (0.21) N 24 G
4′-3-PMA	43.6	2.5	G 55 S 85 (0.05) N 115 (0.17) I	I 110 (0.16) N 75 (0.03) S 42 G
**Polyacrylates** **(4′-n-PAC)**	**10^−3^** $\bar{M}$ **_n_**	$\bar{M}$ **_w_/** $\bar{M}$ **_n_**	**Heating**	**Cooling**
4′-11-PAC	15.9	2.0	G 12 K 107 (2.86) S 115 ^c^ (0.80) I	I 107 (0.72) S 37 (1.08) K 3 G
4′-8-PAC	12.2	1.8	G 11 S 66 (0.03) N 83 (0.39) K 99 (0.55) N 104 (0.07) ^c^	I 97 (0.23) N 63 (0.03) S 6 G
4′-6-PAC	14.0	2.0	G 18 N 101 (0.11) I	I 97 (0.10) N 14 G
4′-6-PAC ^b^	14.0	2.0	K 55 (1.09) N 103 (0.06) I	I 97 (0.10) N 14 G
4′-3-PAC	16.3	1.9	G 38 K 59 (0.02) N 81 (0.03) I	I 76 (0.10) N 14 G
**Polysiloxanes** **(4′-n-PS)**	**10^−3^** $\bar{M}$ **_n_**	$\bar{M}$ **_w_/** $\bar{M}$ **_n_**	**Heating**	**Cooling**
4′-11-PS	13.4	1.8	K 127 (3.81) S 138 (1.20) ^d^ I	I 131 (0.76) S 90 (3.75) K
4′-8-PS	16.4	2.0	K 128 (4.46) I	I 117 (0.22) N 89 (3. 54) K
4′-6-PS	19.6	1.9	G 10 K 124 (3.95) I	I 103 (0.09) N 81 (3.09) K
4′-3-PS	14.3	2.0	G 22 K 84 (1.43) N 110 (0.12) I	I 107 (0.10) N 65 (1.33) K

^a^ Obtained from first heating and cooling scans; ^b^ first heating and cooling scans; ^c^, ^d^ overlapped transitions; G: glass state; N: nematic phase; S: smectic A phase; I: isotropic phase.

**Table 3:** Phase transition behaviors of PSC-11 and PMC-11 [100].

Compounds	GPC		Phase Transitions(°C) and Corresponding Enthalpy Changes (kcal/mru^−1^)	
	$\bar{M}$_n_	$\bar{M}$_w_/$\bar{M}$_n_	Heating	Cooling
PSC-11	5700	1.3	G 50 S1 106 (0.60) S2 168 (0.80) N 216 (0.18) I	I 208 (0.18) N 159 (0.86) S2 88 (0.64) S1
PMC-11	14,600	2.8	G 50 S 129 (0.54) N 166 (0.37) I	I 160 (0.39) N 123 (0.57) S 49 G

G: glass state; N: nematic phase; S: smectic phase; I: isotropic phase.

**Table 4:** Thermal transitions and phase-transition enthaplies for polysiloxanes and polymethacrylates [104].

Spacer Length (n)	Polysiloxanes	Polymethacrylates
	Phase Transitions/°C (Enthalpies/kcal mru^−1^) Heating/Cooling	Phase Transitions/°C (Enthalpies/kcal mru^−1^) Heating/Cooling
3	G 25 S_A_ 117 (1.82) I	K 81 (6.65) S_B_ 86 (0.58) S_A_ 147 (1.07) I
	I 99 (1.80) S_A_	I 145 (1.01) S_A_ 85 (0.55) S_B_ 34 (4.08) K
4	G 18 S_A_ 84 (0.78) I	
	I 80 (0.42) S_A_	
5	G 22 S_B_ 99 (0.89) S_A_ 212 (0.86) I	
	I 204 (0.68) S_A_ 89 (0.82) S_B_	
6	G 16 S_B_120 (1.73) S_C*_ 166 (–) ^a^ S_A_ 244 (1.24) I	K 101 (1.97) S_B_ 123 (0.58) S_A_ 149 (0.82) I
	I 236 (0.98) S_A_ 164 (–) ^a^ S_C*_ 109 (1.47) S_B_	I 142 (0.97) S_A_ 119 (0.53) S_B_ 96 (2.00) K
11	G 20 S_B_109 (0.78) S_C*_ 143 (–) ^a^ S_A_ 218 (0.86) I	K 88 (1.45) S_C*_ 125 (–) ^a^ S_A_ 169 (0.66) I
	I 210 (0. 89) S_A_ 142 (–) ^a^ S_C*_ 103 (0. 74) S_B_	I 165 (0.61) S_A_ 120 (–) ^a^ S_C*_ 81 (0.63) S_B_ 65 (0.07) K

^a^ Enthalpy is very small; G: glass state; S_A_: smectic A phase; S_B_: smectic B phase; S_C*_: chiral smectic C phase; I: isotropic phase.

**Table 5:** Transition temperatures and polymer parameters [105].

Polymers	Transition Temperatures/°C (Enthalpies/J g^−1^)	Molecular Weight ($\bar{M}$_n_)	Polydispersity (γ)
PS	G 22.6 Ch 123.8 (0.27) I	16,300	1.30
PA	G 30.1 Ch 110.8 (0.36) I	9250	1.35
PMA	G 37.8 Ch 101.5 (0.18) I	18,900	1.66

G: glass state; Ch: cholesteric phase; I: isotropic phase.

**Table 6:** Chemical structures of the MJLCPs based on polysiloxane backbones and polyethylene backbone [106].

Conventional MJLCPs.	Chemical Structures	Mesogen-Jacketed Polyailoxanes	Chemical Structures
PMPCS	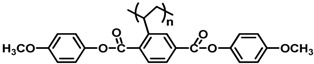	Si-MPCS	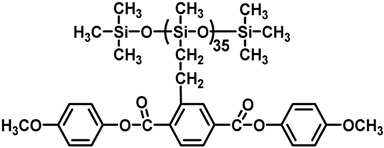
PBPCS	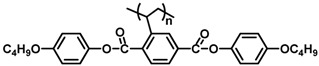	Si-BPCS	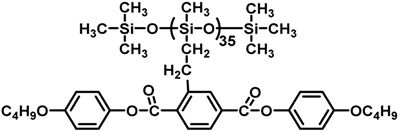
OXD-C12p	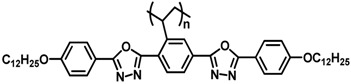	Si-OXD-C12p	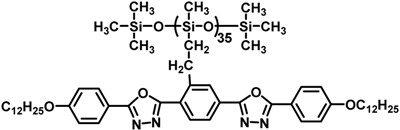
4C2Vp	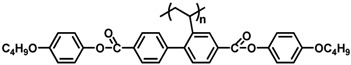	Si-4C2Vp	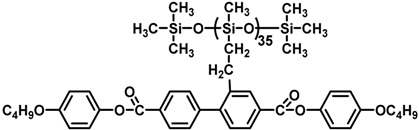
12C2Vp	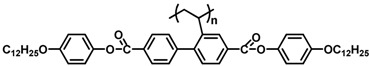	Si-12C2Vp	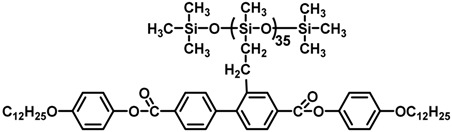

**Table 7:** Comparison of the physical data of the MJLCPs based on polysiloxane backbones and polyethylene backbone [106,107,108,109,110].

Polymers	Molecular Weight ($\bar{M}$_n_)	*T*_g_ (°C)	LC Phase	*d*-Spacing
PMPCS	15,900	118	Φ_N_	1.59
PBPCS	12,700	98	Φ_HN_	1.87
OXD-C12p	142,000	167	S_A_	3.44
4C2Vp	83,000	105	S_A_	3.00
12C2Vp	80,000	86	S_C_	4.20
Si-MPCS	16,600	111	Φ_N_	1.58
Si-BPCS	19,500	92	Φ_HN_	1.86
Si-OXD-C12p	29,200	156	S_C_	3.53
Si-4C2Vp	22,200	96	No	No
Si-12C2Vp	30,000	76	S_A_	4.24

Φ_N_: columnar phase; Φ_HN_: hexagonal columnar phase; S_A_: smectic A phase; S_C_: smectic C phase.

**Table 8:** Thermal transition behaviors of the polysixanes with fluorene unit [112,113].

Polymers	Transition Temperatures/°C Heating/Cooling
1a	G 130–140 S 170–191 I
	I 180–157 S_A_ 142–138 S_E_ 135–116 G
1b	G 137–143 S 174–194 I
	I 192–166 S_A_ 139–130 G
2a	G 141–153 S_E_ 153–158 S_A_ 168–186 I
	I 177–155 S_A_ 154–147 S_E_ 143–133 G
2b	G 97–133 S_E_ 152–163 S_A_ 168–194 I
	I 182–163 S_A_ 153–141 S_E_ 121–80 G

G: glass state; S: smectic phase; S_A_: smectic A phase; S_E_: smectic E phase; I: isotropic phase.

**Table 9:** Thermal transitions and thermodynamic parameters for polysiloxanes with varying degree of polymerization [102].

DP (NMR)	$\bar{M}$_w_/${\bar{M}}_{n}\;(\mathrm{GPC})$	Phase Transitions (°C) and Corresponding Enthalpy Changes (kcal/mru^−1^)	
		Heating	Cooling
7	1.17	G -5 S 10 (0.12) S 72 (1.90) I	I 64 (1.90) S 6 (0.10) S
15	1.14	G 9 S 25 (0.16) S 93 (2.10) I	I 85 (2.00) S 20 (0.15) S
22	1.17	G 10 S 24 (0.15) S 98 (2.00) I	I 87 (2.00) S 16 (0.13) S
27	1.45	G 10 S 21 (0.11) S 95 (1.80) I	I 85 (1.80) S 13 (0.10) S
43	1.60	G 10 S 21 (0.10) S 99 (1.90) I	I 90 (1.80) S 15 (0.09) S
54	1.63	G 10 S 21 (0.08) S 98 (1.60) I	I 87 (1.50) S 16 (0.09) S
56	1.81	G 10 S 21 (0.13) S 98 (1.80) I	I 90 (1.70) S 14 (0.10) S
92	1.72	G 10 S 24 (0.11) S 104 (1.80) I	I 94 (1.70) S 17 (0.12) S

G: glass state; S: smectic phase; I: isotropic phase.

**Table 10:** Thermal transitions and thermodynamic parameters for copolysiloxanes with varying composition [102].

Structural Units Containing Side Groups		(GPC)		Phase Transitions (°C) and Corresponding Enthalpy Changes (kcal/mru^−1^)	
mol % ^a^	wt % ^a^	$\bar{M}$_w_	$\bar{M}$_w_/$\bar{M}$_n_	Heating	Cooling
23.3	65	26,800	2.95	G1 −98 G2 −8 S −7 (0.16) S 37 (1.30) I	I 31 (1.30) S −14 (0.12) S
38.0	79	21,400	2.02	G1 −81 G2 −8 S −6 (0.22) S 47 (1.30) I	I 41 (1.20) S −14 (0.21) S
56.0	89	25,900	2.17	G1 −66 G2 5 S 9 (0.23) S 75 (1.80) I	I 67 (1.80) S 2 (0.19) S
82.0	97	22,800	1.49	G 8 S 18 (0.24) S 92 (2.00) I	I 83 (2.00) S 13 (0.26) S
100.0	100	25,500	2.10	G 8 S 20 (0.13) S 90 (1.80) I	I 81 (1.70) S 13 (0.12) S

^a^ determined by NMR; G: glass state; S: smectic phase; I: isotropic phase.

**Table 11:** Phase behaviors comparison of tetramers [121,122].

Linear Polysiloxane Tetramers	Phase Transitions ^a^ (°C)	PMMS-Based Polysiloxane Copolymers	Phase Transitions ^a^ (°C)
	Heating		Heating
	Cooling		Cooling
PS4-*X*_chol_-1.00	G55.63SmA234.94I	PMMS-X_chol_-1.00	G35.7SmA_d_139.9SmA_s_204.8I
	I234.13SmA56.74G		I197.9SmA_s_136.65SmA_d_34.6G
PS4-*X*_chol_-0.90	G49.94SmA231.35I	PMMS-X_chol_-0.90	G35.6SmA_d’_59.6SmAd152.2SmA_s_189.9I
	I230.32SmA56.51G		I185.5SmA_s_154.9SmAd59.0SmA_d_’34.1G
PS4-*X*_chol_-0.80	G45.10SmA206.39I	PMMS-X_chol_-0.80	G31.9SmA_d_145.1SmA_s_174.4I
	I206.98SmA43.35G		I171.8SmA_s_144.6SmA_d_27.8G
PS4-*X*_chol_-0.70	G50.75SmA187.32Ch213.48I	PMMS-X_chol_-0.70	G30.3SmA_d_164.4I
	I211.63Ch187.25SmA45.37G		I161.5SmA_d_28.9G
PS4-*X*_chol_-0.60	G42.62SmA141.65Ch185.21I	PMMS-X_chol_-0.60	G22.8SmA_d_132.4I
	I183.69Ch141.39SmA39.57G		I129.8SmA_d_22.6G
PS4-*X*_chol_-0.50	G40.51Ch178.89I	PMMS-X_chol_-0.50	G21.9SmA_d_114.7I
	I177.38Ch39.01G		I117.7SmA_d_20.1G
PS4-*X*_chol_-0.40	G34.48Ch156.72I	PMMS-X_chol_-0.40	G20.2SmA_s_110.7I
	I155.20Ch32.59G		I108.3SmA_s_18.6G
PS4-*X*_chol_-0.30	G24.00Ch106.50I	PMMS-X_chol_-0.30	G16.0SmE55.9SmA_s_102.5I
	I105.40Ch23.00G		I99.83SmA_s_5I.3SmE11.8G
PS4-*X*_chol_-0.20	G18.39Ch82.50I	PMMS-X_chol_-0.20	G7.9SmE70.6SmA_s_80.0I
	I81.42Ch15.99G		I72.5SmA_s_63.4SmE9.6G
PS4-*X*_chol_-0.10	G11.41Ch64.06I	PMMS-X_chol_-0.10	G11.4SmE’46.8SmE71.9SmA_s_90.0I
	I62.53Ch8.98G		I76.5SmA_s_71.9SmE57.4SmE’8.91G
PS4-*X*_chol_-0.00	G6.97N32.09I	PMMS-X_chol_-0.00	G17.1SmE83.4SmA_s_95.5I
	I30.04N4.80G		I80.5SmA_s_68.6SmE15.2G

^a^ According to the DSC results; G: glass state; Ch: cholesteric phase; SmA: smectic A phase; SmE: Smectic E phase; SmE’: Smectic E phase with microphase separation; SmA_s_: smectic A phase with a single layer; SmA_d_: smectic A phase with a double layer; SmA_d_’: SmA_d_ phase with microphase separation; I: isotropic state.

**Table 12:** Interlayer distance d (nm) and the phase transition temperatures as a function of the number of siloxane units [123].

x	*d* (nm)	Phase Transitions (°C)
4	3.13	K 50 S 118 I
5	3.16	K 44 S 116 I
6	3.15	K 43 S 124 I
7	3.26	K 37 S 119 I
8	3.27	K 38 S 118 I

K: crystal; S: smectic phase; I: isotropic phase.

**Table 13:** Phase behaviors of the homopolymers and copolymers [125].

5R		4R	
Compound ^a^	Thermal Transitions (°C)	Compound ^a^	Thermal Transitions (°C)
5R1	G 66 N 135 I	4R1	G 110 Viscous melt
5R2	K_1_ 115 K_2_ 130 N 175 I	4R2	K 180 N 200 I
5R3	K 148 N 175 I I 172 N 140 S_C_ 120 K	4R3	K 188 I I 178 N 148 K
5R4	K_1_ 80 K_2_ 103 S_A_ 172 I	4R3	K 116 S_A_ 169 I
5R5	G 76 S_A_ 247 I	4R5	G 100 S_A_ 232 I
5R6	G 61 S_A_ 228 N* 246 I	4R6	G 76 S_A_ 270 N* 277 I
5R7	G 50 S_A_ 255 I	4R7	G 58 S_A_ 263 I
5R8	G 49 S^*^_C_ 100 S_A_ 252 I	4R8	G 55 S^*^_C_ 100 S_A_ 246 I
5R1/5	G 80 N* 204 I	4R1/5	G 75 N* 204 I
5R2/6	G 50 N* 220 I	4R2/6	G 57 N* 237 I
5R3/7	G 41 S^*^_C_ 130 S_A_ 198 N* 228 I	4R3/7	G 35 N* 220 I
5R4/8	G 26 S_A_ 199 N* 209 I	4R4/8	G 30 S_A_ 204 N* 210 I

^a^ 5R and 4R refer to the ring size, the last number corresponds to mesogen attached (see Figure 14). G: glass state; K: crystal; S_A_: smectic A phase; S^*^_C_: chiral smectic C phase; N*: chiral nematic phase; I: isotropic phase.

**Table 14:** Phase behaviors comparison of the tetramers [122,132].

Linear Polysiloxane Tetramers	Phase Transitions ^a^ (°C)	Cyclic Polysiloxane Tetramers	Phase Transitions ^a^ (°C)
	Heating		Heating
	Cooling		
PS4-*X*_chol_-1.00	G55.63SmA234.94I	PCS4-X_chol_-1.00	G65.5SmA233.1Ch248.0BPs266.5I
	I234.13SmA56.74G		
PS4-*X*_chol_-0.90	G49.94SmA231.35I	PCS4-X_chol_-0.875	G56.4SmA220.2Ch245.4BPs251.0I
	I230.32SmA56.51G		
PS4-*X*_chol_-0.80	G45.10SmA206.39I	PCS4-X_chol_-0.75	G657.3SmA197.7Ch237.5BPs242.5I
	I206.98SmA43.35G		
PS4-*X*_chol_-0.70	G50.75SmA187.32Ch213.48I	PCS4-X_chol_-0.625	G55.2Ch230.5BPs234.5I
	I211.63Ch187.25SmA45.37G		
PS4-*X*_chol_-0.60	G42.62SmA141.65Ch185.21I	PCS4-X_chol_-0.50	G43.4Ch205.2BPs208.0I
	I183.69Ch141.39SmA39.57G		
PS4-*X*_chol_-0.50	G40.51Ch178.89I	PCS4-X_chol_-0.375	G53.7Ch208.3I
	I177.38Ch39.01G		
PS4-*X*_chol_-0.40	G34.48Ch156.72I	PCS4-X_chol_-0.25	G51.5Ch192.7I
	I155.20Ch32.59G		
PS4-*X*_chol_-0.30	G24.00Ch106.50I	PCS4-X_chol_-0.125	G55.5Ch189.2I
	I105.40Ch23.00G		
PS4-*X*_chol_-0.20	G18.39Ch82.50I	PCS4-X_chol_-0	Cr_1_45.6Cr_2_156.9N194.4I
	I81.42Ch15.99G		
PS4-*X*_chol_-0.10	G11.41Ch64.06I		
	I62.53Ch8.98G		
PS4-*X*_chol_-0.00	G6.97N32.09I		
	I30.04N4.80G		

^a^ According to DSC results; G: glass state; Ch: cholesteric phase; SmA: smectic A phase; BP: blue phase; Cr: crystal; I: isotropic state.

**Table 15:** Phase transition behaviors of homo-polysiloxanes and co-polysiloxanes containing end-on fixed mesogenic side groups [150].

Polymers	$\bar{M}$_n_ × 10^3^ (GPC)	$\bar{M}$_w_/$\bar{M}$_n_ (GPC)	Thermal Transitions/°C and Corresponding Enthalpy Changes (kcal mru^−1^) in Parentheses ^a^	
			Heating	Cooling
P-11a-2	2.7	1.51	G18K52(0.98)S_A_298(0.72) ^b^ I	I268(0.95)S_A_16G
P-11a-2/3-2(8/2)	4.9	1.51	K51(1.56)S_A_235(0.41)I	I224(0.49)S_A_10G
			g 7 S_A_ 231 (0.45) I	
P-11a-2/3-2(6/4)	6.1	1.84	G-2K41(0.64)S_A_158(0.05) I	I156(0.33)S_A_-10G
			G-6S_A_159(0.20)I	
P-11a-2/3-2(4/6)	9.5	1.86	K40(0.82)S_A_92(0.02) ^c^ I	I92(0.13) ^c^ S_A_-21G
			G-19K31(0.02)S_A_95(0.01) ^c^ I	
P-11a-3	3.0	1.12	K51(2.20)S_A_265(0.94)I	I257(0.95)S_A_5G
			G10SA264(0.86)1	
P-11a-3/3-3(8/2)	3.3	1.07	K47(1.58)S_A_204(0.37)I	I190(0.26)S_A_-11G
			G-5K41(0.03)S_A_195(0.45)I	
P-11a-3/3-3(6/4)	3.5	1.10	K36(0.91)S_A_105(0.05) ^c^ I	I107(0.14) ^c^ S_A_-20G
			G-16K26(0.08) ^d^ K34(0.10)S_A_108(0.05) ^c^ I	
P-11b-2	14.4	1.97	K57(0.46)K121(1.98)S_A_164(0.18)N212(0.35)I	I209(0.20)N157(0.15)S_A_92(2.03)K68(0.34)K
			K76(0.39)K123(2.32)S_A_163(0.15)N213(0.22)I	
P-11b-3	10.4	2.00	K64(0.40)K107(2.00)N160(0.18)I	I155(0.16)N83(1.79)K52 (0.40)K
			K63(0.38)K107(1.98)N159(0.16)I	

K: crystal; G: glass state; S_A_: smectic A phase; I: isotropic state. ^a^ Data in the first line are from the first heating and cooling scans, data in the second line are from the second heating scan; ^b^ decomposition; ^c^ very broad transition (enthalpy change could not be accurately determined); ^d^ crystallization exotherm.

**Table 16:** Phase transition behaviors of P-11a-3-Li^+^CF_3_SO_3_ complexes [150].

Mole of Li^+^CF_3_SO_3_^−^ Per Mole Repeat of P-11a-3	Thermal Transitions/°C and Corresponding Enthalpy Changes (kcal mru^−1^) in Parentheses ^a^	
	Heating	Cooling
0	K51(2.20)S_A_287 ^b^ I	S_A_5I
	G10S_A_287 ^b^ I	
0.3	G15S_A_270 ^b^ I	S_A_18I
	G23S_A_270 ^b^ I	
0.7	K48K55(1.13) ^c^ S_A_237 ^b^ I	S_A_30I
	G36S_A_237 ^b^ I	
1.1	K53(0.65)S_A_234 ^b^ I	S_A_29I
	G38S_A_234 ^b^ I	
1.7	K47K58(0.44) ^c^ S_A_231 ^b^ I	S_A_39I
	G45S_A_231 ^b^ I	
2.0	K48K57K65(0.83) ^c^ S_A_228 ^b^ I	S_A_40I
	G45S_A_228 ^b^ I	
2.5	K58(0.16)K66(0.43)S_A_222 ^b^ I	S_A_41I
	G44S_A_222 ^b^ 1	
2.7	K56(0.31)K64(0.34)S_A_ 220 ^b^ I	S_A_38I
	G44S_A_220 ^b^ I	
3.0	K56(0.31)K64(0.21)S_A_221 ^b^ I	S_A_45I
	G46S_A_221^b^I	
3.3	K55(0.35)K64(0.21)S_A_215 ^b^ I	S_A_44I
	G47S_A_215 ^b^ I	
4.5	K55K64(0.59) ^c^ S_A_ 14 ^b^ I	S_A_41I
	G43S_A_214 ^b^ I	

K: crystal; G: glass state; S_A_: smectic A phase; I: isotropic state. ^a^ Data in the first line are from the first heating and cooling scans, data in the second line are from the second heating scan; ^b^ Determined by optical polarized microscopy; ^c^ Overlapping transitions (enthalpy change is the total of both transitions).

**Table 17:** Phase transition behaviors of homo-polysiloxanes containing side-on fixed mesogenic side groups [151].

Polymers	$\bar{M}$_n_ × 10^3^ (GPC)	$\bar{M}$_w_/$\bar{M}$_n_ (GPC)	Thermal Transitions/°C and Corresponding Enthalpy Changes (kcal mru^−1^) in Parentheses ^a^	
			Heating	Cooling
P-12-1	7.8	1.97	G41K88(0.29)N122(0.13)I	I125(0.19)N47G
			G50N129(0.18)I	
P-12-2	9.7	1.27	K54(0.45)K90(2.33)N109(0.31)I	I107(0.20)N35G
			G39N111(0.19)I	
P-12-3	12.0	1.84	K53(0.21)K83(2.99)N104(0.17)I	I98(0.19)N26G
			G31N103(0.23)I	

K: crystal; G: glass state; N: nematic phase; I: isotropic state. ^a^ Data in the first line are from the first heating and cooling scans, data in the second line are from the second heating scan.

**Table 18:** Phase transition behaviors of P-12-3/Li^+^CF_3_SO_3_^−^ complexes [151].

Mole of Li^+^CF_3_SO_3_^−^ Per Mole Repeat of P-12-3	Thermal Transitions/°C and Corresponding Enthalpy Changes (kcal mru^−1^) in Parentheses ^a^	
	Heating	Cooling
0	K53(0.21)K83(2.99)N104(0.17)I	I98(0.19)N26G
	G31N103(0.23)I	
0.1	K70(3.68)I	I67(0.06)N16G
	G19N71(0.07)I	
0.2	K66(3.16)I	I60(0.07)N20G
	G22N 65(0.05)	
0.3	K60(2.67)I	I55(0.06)N20G
	G26N61(0.05)	
0.4	K60(1.87)I	I48(0.05)N24G
	G28N54(0.06)I	
0.5	K45(0.87)I	I26G
	G28I	
0.6	K48(0.35)I	I31G
	G31I	
0.7	- ^b^	I33G
	G33I	
0.8	- ^b^	I43G
	G48I	

K: crystal; G: glass state; N: nematic phase; I: isotropic state. ^a^ Data in the first line are from the first heating and cooling scans, data in the second line are from the second heating scan; ^b^ No glass transition or melting endotherm could be detected.

**Table 19:** Physical properties of the cyanobiphenyl-based polysiloxanes containing long thioether spacers (first heating) [154].

Polymers (P_n,m_)	*T*_g_/°C	*T*_k_/°C	*T*_i_/°C	*ΔT*/°C		Mesophase
				(1)	(2)	
P_6,2_	−15	-	101	116	-	SmA
P_8,2_	−20	-	118	138	-	SmA
P_10,2_	−21	-	125	146	-	SmA
P_10,3_	−10	-	135	145	-	SmA
P_11,2_	−22	45	121	-	76	SmA
P_11,3_	−15	68	140	-	72	SmA
P_11,-_	-	52	181	-	129	SmA

SmA: smectic A phase; *T*_g_: Glass transition temperature; *T*_k_: Melting temperature (from crystallization); *T*_i_: Isotropisation temperature; *ΔT* = *T*_i_ − *T*_g_ (1); *ΔT* = *T*_i_ − *T*_k_ (2).

**Table 20:** Mesomorphic properties of the polysiloxane-based LCPs with thioether spacer via thiol−ene click chemistry [39].

Polymers	Phase Transitions (°C) ^a^	
	Heating	Cooling
PMMS-g-LC1	SmX 62 I	I 59 SmX
PMMS-g-LC2	SmX 82 I	I 80 SmX
PMMS-g-LC3	N 70 I	I 68 N
PMMS-g-LC4	N73 I	I 71 N
PMMS-g-LC5	N 56 I	I 54 N

^a^ Detected only by polarized optical microscopy. N = nematic, SmX = undefined smectic phase, I = isotropic, G = glass phase.

**Table 21:** Mesomorphic properties of PMMS-CN-based polymers [90].

Polymers	*X*_chol_^a^		*M*_n_ (g/mol)		Phase Transitions (°C) ^d^	
	In Feed	CALC ^b^	GPC ^c^	CALC ^b^	Heating	Cooling
P1	0.00	0.000	2200	18,747	G2.4SmA_s_92.1I	I81.3SmA_s_-1.4G
P2	0.20	0.221	3300	21,412	G10.5SmA_s_96.3 I	I98.5SmA_s_8.8G
P3	0.40	0.425	3700	23,872	G18.7SmA_s_111.7I	I113.4SmA_s_16.4G
P4	0.60	0.604	4000	26,031	G23.6SmA_s_176.8I	I175.8SmA_s_19.5G
P5	0.80	0.819	4400	28,624	G33.1SmA_s_189.4I	I186.7SmA_s_29.9G
P6	1.00	1.000	4800	30,852	G35.7SmA_d_139.9SmAs204.8I	I197.9SmA_s_136.6SmA_d_34.6G

^a^ Molar ratio; ^b^ According to ^1^H-NMR results; ^c^ determined by GPC in THF using polystyrene standards; ^d^ according to DSC results; G: glass state; SmA_s_: smectic A phase with single layer; SmA_d_: smectic A phase with double layer; I: isotropic state.
